# Milling-Induced Defects in Ni/Zirconia Catalysts for Enhancing Catalytic Activity in Dry Methane Reforming

**DOI:** 10.1021/acs.jpcc.5c08218

**Published:** 2026-03-04

**Authors:** Joanna Elzbieta Olszowka, Volodymyr Sydorchuk, Karolina Simkovicova, Mehran Sajad, Guillaume Clet, Michal Horacek, Graham King, Jan Pasztor, Stefan Vajda

**Affiliations:** † Department of Nanocatalysis, 86875 J. Heyrovsky Institute of Physical Chemistry, Czech Academy of Sciences, Prague 182 00, Czech Republic; ‡ 27003 Université de Caen Normandie, ENSICAEN, CNRS, LCS, Caen 14000, France; § Department of Molecular Electrochemistry and Catalysis, J. Heyrovsky Institute of Physical Chemistry, Czech Academy of Sciences, Prague 182 23, Czech Republic; ∥ 117197 Canadian Light Source, Saskatoon S7N 2 V3, Canada; ⊥ NICOLET CZ, Prague 149 00, Czech Republic

## Abstract

Alteration of the support structure via milling is a feasible yet rarely applied strategy for boosting the performance of the catalyst in dry methane reforming for syngas production. In this study, we introduce stable oxygen vacancies in the zirconia structure, which enhance the activation of the feedstock, specifically CO_2_, while preserving the specific surface area and porosity of the material under reaction conditions. The activity of the tested Ni/ZrO_2_ assemblies shows a clear dependence on the milling intensity of ZrO_2_, with mild milling at 400 rpm yielding the most active catalyst. At 600 °C, this material achieved the highest feedstock conversion among the tested samples, with 29% for CH_4_ and 39% for CO_2_. Spectroscopic characterization indicates that the activity of the tested catalysts is controlled by a partial change in the phase composition of the support from monoclinic to tetragonal under reaction conditions, as well as the nature and population of O_2_^–^ species, oxygen vacancies, Zr^3+^ defects, and Ni–ZrO_2_ interfacial interactions.

## Graphical abstract

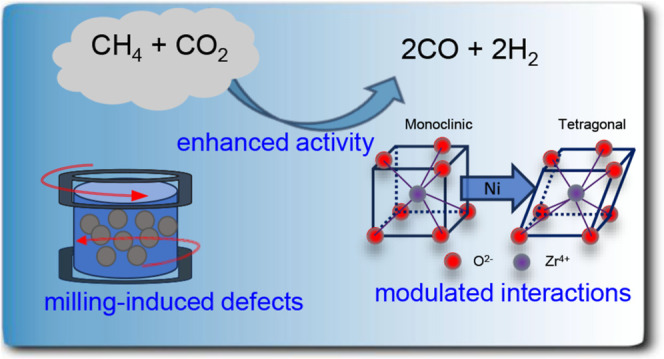

## 1 Introduction

Zirconium oxide (ZrO_2_) is commonly applied as a heterogeneous catalyst and support material in many important processes encompassing red-ox, acid–base, and photocatalytic reactions.^1–3^ Among them, its use in dry methane reforming (DMR) has been extensively studied to convert two greenhouse gases, CO_2_ and CH_4,_ simultaneously. For this reaction, ZrO_2_ is reported as a promising support for transition metal nanoparticles, particularly nickel,^4,5^ due to its high thermal stability, redox properties, and enhanced metal–support interactions.^2,6,7^ Moreover, ZrO_2_ supports enhance Ni dispersion, oxygen storage capacity, and surface basicity, thereby promoting efficient CO_2_ activation and facilitating the continuous removal of carbonaceous species during DMR.^5^ In particular, enhanced CO_2_ activation at the Ni–ZrO_2_ interface increases the availability of reactive oxygen species, formed via CO_2_ dissociation together with CO, which can react with carbon deposits formed predominantly from CH_4_ decomposition,^8–10^ improving resistance to coke formation under high-temperature conditions.^11^ Oxygen vacancies are key defect sites in reducible oxides that enhance CO_2_ activation during dry methane reforming. The resulting oxygen species can participate in subsequent oxidation of carbonaceous intermediates, helping to mitigate coke formation and sustain catalytic performance under DMR conditions.^12^ In addition, phase engineering of ZrO_2_, especially stabilization of tetragonal or mixed zirconia phases, can strengthen metal–support interactions. This effect is closely related to the presence of oxygen vacancies, which not only contribute to catalytic activity but also stabilize the thermodynamically metastable tetragonal phase.^9,13–15^ Conventionally, tuning these structural features is achieved by adjusting synthesis parameters during precipitation or through postsynthesis treatments such as pH control and calcination.

Because DMR operates at elevated temperatures, the support must exhibit exceptional thermal stability while maintaining its physicochemical properties. Traditional preparation methods for ZrO_2_ and Ni/ZrO_2_ catalysts (e.g., precipitation, sol–gel synthesis, impregnation) require high-temperature calcination to generate crystalline ZrO_2_ and NiO phases and to introduce oxygen vacancies. However, such treatments often lead to a significant decrease in specific surface area (SSA) due to partial sintering and pore collapse, accompanied by increased NiO crystallite size and decreased reducibility. As a result, Ni/ZrO_2_ catalysts prepared by conventional routes typically exhibit SSA values in the range of 15–40 m^2^ g^–1^,^5,16,17^ whereas plasma-assisted or hydrothermal approaches can provide surface areas exceeding 50 m^2^ g^–1^.^8,14,18^ Preserving high surface area, preventing phase transitions, and minimizing sintering of both the support and the deposited metal nanoparticles are therefore essential for achieving long-term catalytic stability.

Hydrothermal treatment (HT) offers an effective route to obtain highly crystalline monoclinic ZrO_2_ with a well-developed mesoporous structure and negligible microporosity.^7,18^ It was shown that treatment of precipitated ZrO_2_ at 260–320 °C promotes the formation of a pure monoclinic phase, whose crystallinity and surface properties can subsequently be modified by ball milling.^18^ This mechanochemical approach is attractive from both technological and environmental perspectives, enabling substantial reductions in waste generation and energy consumption compared to conventional synthesis routes.^19^ Hydrothermally treated samples retain a uniform mesoporous structure with enhanced thermal stability, and this structure is largely preserved during subsequent mild milling. Importantly, soft postmilling of hydrothermally prepared monoclinic ZrO_2_ in air enables the introduction of structural defects, most likely oxygen vacancies, without altering the phase composition.^18^ Previous UV–Vis and photocatalytic studies have indicated that these defects remain stable even after postcalcination.^18^ Nevertheless, despite its simplicity, this strategy for defect engineering in oxide supports has not yet been systematically explored in the context of thermal catalysis for DMR, although recent reviews have identified mechanochemical treatment as a promising route for generating oxygen vacancies.^20,21^ Furthermore, the stability of milling-induced oxygen vacancies is not guaranteed, as post-treatment steps such as washing can lead to their quenching.^1,22^ For ZrO_2_ in particular, prior studies of milling have primarily focused on phase transformations rather than catalytic performance.^18,23–26^ The studies on the structural evolution of milled ZrO_2_ under reaction conditions, especially the monoclinic-to-tetragonal transformation and the behavior of oxygen vacancies, remain scarce.^4,27^ Therefore, building upon earlier findings, we employed a combined hydrothermal-milling approach as a strategy for synthesizing thermally stable, defect-engineered ZrO_2_ supports with boosted activity.

The main objectives of this work are to identify the nature and extent of milling-induced defects in ZrO_2_ support materials and to assess their stability, enabling us to draw a relationship between the structural characteristics of the modified zirconias and the performance of Ni catalysts prepared with these altered supports in dry methane reforming. Our results demonstrate a rarely explored yet highly effective strategy for catalyst design: tuning the zirconia support through controlled milling to introduce stable oxygen vacancies while preserving its textural properties. By deliberately engineering the defect landscape of ZrO_2_, we achieve a targeted enhancement of CO_2_ adsorption and activation, which can, in turn, contribute to more efficient carbon removal and mitigation of coke formation during reaction. This approach opens a new route for enhancing syngas catalyst performance through support modification rather than conventional metal-centered optimization, providing a conceptually distinct and scalable strategy for catalyst development.

## 2 Experimental Section

### 2.1 Reagents

The zirconium oxynitrate hydrate, ZrO­(NO_3_)_2_·2H_2_O (99.99% trace metals basis), and a 25% aqueous ammonia solution purchased from Merck were used for the precipitation of hydrous zirconium oxide. Nickel­(II) oxide, NiO (99.8% trace metals basis), was purchased from Merck in the form of nanopowder (<50 nm particle size). Commercial zirconia (nanopowder) purchased from Merck was used as a reference.

### 2.2 Precipitation

The detailed precipitation of ZrO_2_ gel is described in our previous paper.^18^ An aqueous solution of ammonium hydroxide (11.5 M) was added dropwise to a 0.35 M ZrO­(NO_3_)_2_ aqueous solution with vigorous stirring until pH 7 was reached. This pH value was chosen because a pure monoclinic phase is formed upon subsequent hydrothermal treatment of the obtained precipitated ZrO_2_. The resulting gel was kept in the mother solution for 24 h at room temperature. Then, the gel was separated and washed with distilled water by decantation until the pH value of the rinsing water was neutral. The wet gel was filtered and dried for 48 h at room temperature to form a dried xerogel.

### 2.3 Modification Procedures

The resulting ZrO_2_ xerogel was subjected to hydrothermal treatment at a temperature of 300 °C for 7 h. Hydrothermal treatment (HT) was performed using a Teflon-lined steel autoclave with a volume of 45 mL. The xerogel was loaded into a quartz tube and placed in an autoclave. Fifteen ml of water was added to the bottom of the autoclave to create saturated vapor pressure.

The hydrothermally modified xerogels were subjected to dry milling without the addition of liquid media (in air) at 400–500 rounds per minute (rpm) for 30 min in two cycles, each consisting of 15 min with a 5 min pause and reverse. Milling was performed using a Pulverisette-7 planetary ball mill, premium line (Fritsch GmbH), with an 80 mL zirconia vessel to minimize possible contamination with other materials. The ratio of the mass of the balls to the mass of the sample (ball-to-powder, BPR) was equal to 10.1. As working bodies, zirconium dioxide balls with a diameter of 5 mm and a total mass of 91.5 g were used, and the mass of the sample was about 9.1 g. The properties of the materials obtained with the repetition of the described preparation protocol were reproducible.

The list of prepared samples with their designations is presented in Table 1, where HT and M denote hydrothermal treatment and milling, respectively; the numbers show the intensity of milling in the form of rpm (rotations per minute).

**Table 1:** List of Samples with Designations and Conditions of Preparation

designation	conditions of modification	designation after Ni addition
ZrO_2__HT	HT xerogel 300 °C 7 h	Ni/ZrO_2__HT
ZrO_2__HT_M400	HT xerogel 300 °C 7 h + milling 400 rpm 0.5 h	Ni/ZrO_2__HT_M400
ZrO_2__HT_M450	HT xerogel 300 °C 7 h + milling 450 rpm 0.5 h	Ni/ZrO_2__HT_M450
ZrO_2__HT_M500	HT xerogel 300 °C 7 h + milling 500 rpm 0.5 h	Ni/ZrO_2__HT_M500
ZrO_2__com	commercial ZrO_2_ nanopowder	-

The zirconia supports obtained were finally intimately mixed in a mortar with commercial NiO to achieve 10 wt % nickel content in the final material for catalytic testing and operando studies. The final uniform physical composite was obtained with no wet impregnation or chemical anchoring.

### 2.4 Physicochemical Characterization

X-ray diffraction (XRD) analysis of support materials was performed using a DRON-3 M diffractometer (CuKα radiation, λ = 0.154 nm). Lattice parameters of the ZrO_2_ phase and microstructural parameters of the samples (average grain size D and microstrain content < *e* >) were derived by complete profile Rietveld refinement using WinCSD software.^28^ Garvie-Nicholson method allowed for determining the molar content of the monoclinic phase *X*_m_ according to the following eq (eq 1)^29^

1 $$X_{m}=\left[I_{m}\left(111\right)+I_{m}\left(-111\right)\right]/\left[I_{m}\left(111\right)+I_{m}\left(-111\right)+I_{t}\left(101\right)\right]$$

where *I*_m_(111) and *I*_m_(−111) denote the intensity of peaks of the monoclinic phase at 28.2° and 31.3°, respectively, and *I*_t_(101) denotes the intensity of the peak of the tetragonal phase at 30.4°.

The morphology of the hydrothermally as-synthesized ZrO_2_ powder and its milled counterparts was characterized with a Hitachi S4800 scanning electron microscope (SEM) equipped with a Nanotrace electron diffraction EDX detector (Thermo Electron).

Synchrotron total scattering data of all support materials (without Ni addition) were collected at the Brockhouse High Energy Wiggler Beamline of the Canadian Light Source using 60.83 keV X-rays. The data was collected with a Varex XRD 4343 CT area detector. The samples were contained in 0.63 mm inner diameter Kapton capillaries. The data was processed and integrated using GSAS-II.^30^ The pair distribution functions (PDF) were generated using a *Q*_max_ of 25 Å^–1^.

The porous structure of support ZrO_2_ samples was characterized using adsorption–structural methods. Nitrogen isotherms of adsorption–desorption were recorded using an automatic gas adsorption analyzer ASAP 2405N (“Micromeritics Instrument Corp”) after outgassing the samples at 150 °C for 20 h. The specific surface area (*S*), volume of mesopores (*V*_me_), and volume of micropores (*V*_mi_) were calculated from these isotherms using the BET, BJH, and t-methods, respectively. Total pore volume (*V*) was determined at a relative pressure of nitrogen *p*/*p*_0_ close to 1 and consists of the volume of micro- and mesopores. The pore size distribution (PSD) curves were plotted using the desorption branches of isotherms.

For the characterization of the support in vacuum, Fourier transform far and mid-infrared (FIR and MIR) spectroscopy measurements at room temperature were conducted with a FTIR spectrometer, a Nicolet iS50 FTIR spectrometer with a diamond attenuated total reflectance crystal. Raman spectroscopy was performed in ambient conditions under vacuum on a Nicolet DXR3 Raman Microscope with a 532 nm excitation laser and 10× and 50× objectives on the same samples at the same conditions.

Electron paramagnetic resonance (EPR) spectra of support materials were recorded on a CW X-band EPR spectrometer MiniScope MS400 (Magnettech) equipped with microwave frequency counter FC 400 and temperature controller TC H03. EPR spectrometer MiniScope MS400 (Magnettech) is an instrument with high measurement sensitivity 8*109 Spins/0.1 mT and magnetic field stability 1.5 μT/min. The 4 mm quartz tubes were filled with solid samples (a white powder prior to the reactions) in order to fill the cuvette volume in the resonance cavity of the EPR spectrometer. After the reactions, the solid samples (white powder) were transferred to Hirschmann capillaries (ring caps, 50 μL), covered with wax, and measured. The amount of sample after the reactions did not fill the resonance cavity of the EPR spectrometer. The EPR spectra of the samples were recorded at −160 °C. The parameters were evaluated using the ESR-Mplot & Analyze interactive EPR data processing program (Magnettech GmbH). Data processing for the figures was performed using OriginPro 2023 software (OriginLab Corporation).

To study in situ the lattice vibrations of ZrO_2_ in the support and Ni-containing samples, diffuse reflectance measurements were performed in the far-Infrared (FIR) region (30–600 cm^–1^) at the Canadian Light Source (CLS) Far-Infrared beamline using a Bruker IFS125HR spectrometer equipped with a T222 Mylar beamsplitter. The infrared beam was directed out of the sample compartment into a Pike Instruments Diffuse IR accessory and then to a QMC Superconducting Niobium Transition-Edge Sensor (TES) bolometer. The synchrotron was utilized due to its high photon flux below 200 cm^–1^. The ZrO_2_ samples were packed into ceramic sample cups and placed in the environmental chamber of the DiffusIR accessory. This chamber, with an internal volume of 63 cm^3^, featured a silicon window. Argon gas continuously flowed through the chamber at a rate of 200 mL/min, controlled by a Brooks SLA5850 mass flow controller. All spectra were collected at a resolution of 4 cm^–1^ and averaged over 5–10 sets of measurements, each consisting of 2048 scans. Data processing was conducted using the OPUS software package to obtain the reflectance spectra. These spectra were produced by dividing the obtained spectra by those of polyethylene or KBr powder in the case of CO_2_ and CH_4_ adsorption. The spectra of the reference powders, which served as a background, were collected immediately before and after the sample spectra under identical conditions. The samples were purged of air moisture in argon and then submitted to conditions comparable with the catalytic testing: reduction in 10% H_2_ in argon for 1 h at 550 °C, switch to reaction mixture consisting of 10% CO_2_ and 10% CH_4_ at the temperatures 550 °C, 600 °C, 650 °C, and then cooled down to 20 °C.

Raman *operando* characterization of Ni-containing samples was performed on a Horiba Labram HR Evolution spectrometer using a 532 nm laser excitation, with analysis of the solids (ca. 40 mg) performed within a Linkam CCR-1000 reaction cell. The prepared ZrO_2_ samples (ZrO_2__HT, ZrO_2__HT_M400, ZrO_2__HT_M450, ZrO_2__HT_M500) were mixed with NiO nanoparticle powder in a mortar to a total w/w concentration of NiO 10%. The first spectra were measured at room temperature. After that, the temperature in the cell was increased to 550 °C with a ramp of 10 °C/min under a flow of H_2_ (4 mL/min) and Ar (36 mL/min) and held at this temperature for 1 h. Raman spectra were recorded for the whole reduction duration. Then the reaction mixture was introduced, which consisted of 2 mL/min CO_2_, 2 mL/min CH_4,_ and Ar at 36 mL/min. The temperature was then increased by 10 °C/min to 650 °C under the reaction gases. The reaction was carried out for at least 30 min at each temperature, and spectra were measured continuously. The sample was then cooled at the rate of 20 °C/min, and spectra were finally recorded on the solid back at 25 °C. For the detection of substrates and products, mass spectrometry was used, which confirmed the production of H_2_ during the reaction.

### 2.5 Catalytic Testing

A microcapillary reactor was utilized for steady-state catalytic measurements of the bare supports and Ni-containing counterpart samples. The reactor consists of a quartz capillary tube (approximately 1 mm outer diameter, 0.4 mm thickness, and 50 mm length, heated by two 30 mm long coil heaters wrapped around a ceramic support positioned in the closest vicinity of the capillary.^31^ The temperature inside the sample was controlled by an electronic temperature controller (Eurotherm 2404) and an ATE 55–10DM Kepco power supply. The temperature was measured using an in-bed thermocouple (type K, Omega) with an accuracy of ±2 °C. The DMR reaction was conducted at a constant preset pressure of approximately 1.2 bar, maintained by a downstream mass flow controller (Brooks SLA5850) integrated into a regulation loop driven by a diaphragm pump (Divac 1.4HV3). The pressure was measured using a transducer (Omega PX209) and kept stable and controlled by custom software written in Python across the applied temperature range (25–650 °C). Catalyst powder weighing 0.3 mg, with a bed length of approximately 1.5 mm, was held between two pieces of porous quartz wool bed within the capillary reactor. The active Ni phase was subsequently generated in situ by reduction of NiO to metallic Ni prior to catalytic testing. First, the catalysts were treated in the flow of 5% H_2_ diluted in argon with a total flow of 10 mL/min, in the temperature range 25 to 550 °C with a heating ramp of 10 °C/min, then maintained at 550 °C for 1.5 h as the reduction treatment. DMR reaction was performed at 550, 600, and 650 °C, where 1% of CH_4_ and 1% of CO_2_ diluted in argon with a total flow of 10 mL/min were introduced through heated transfer lines set at 100 °C. The catalyst was exposed to the reactants for 30 min at each temperature prior to measurement to achieve equilibrium. The flow rates were adjusted using mass flow controllers (Brooks Instruments). The concentrations of reactants and products were determined with gas chromatography, Inficon MicroGC Fusion, equipped with Rt-Molsieve 5A (0.25 mm ID, 10 m), Rt-Q Bond (0.25 mm ID, 12 m), and Rxi-1 ms (0.15 mm ID, 20 m) columns, and thermal conductivity detectors (TCD). For blank tests, the supports, i.e., without the active Ni phase, were subjected to identical conditions, as mentioned above. The effluent stream was also qualitatively analyzed by sampling into the differentially pumped mass spectrometer chamber using an electronic needle control valve (Pfeiffer EVR 116). The flow rate was regulated by a regulator (Pfeiffer RVC 300) and a pressure gauge (Pfeiffer PKR 261) to maintain a constant pressure set to 5.0 × 10^–6^ mbar in the mass spectrometer chamber. The mass spectrometer operated in continuous mass scanning mode (2 scans per minute) over a range of 0 to 100 *m*/*z*, controlled by PV MassSpec software (Pfeiffer). The conversion of the gas feedstock (for each substrate, CH_4_ and CO_2_) and yield of the products (for H_2_ and CO) were calculated using the formulas described in the literature.^32^

## 3 Results

### 3.1 Structure, Morphology, and Porosity of the Obtained Zirconia Supports

#### 3.1.1 XRD Characterization of the Support

As previously reported,^18^ precipitated ZrO_2_ is X-ray amorphous, but further hydrothermal treatment (HT) of the dried xerogel at 300 °C leads to the formation of a well-crystallized pure monoclinic phase (Figure 1, sample ZrO_2__HT). As can be seen in the spectra of ZrO_2__HT_M400, ZrO_2__HT_M450, and ZrO_2__HT_M500 samples, the monoclinic structure is preserved after postmilling.

**Figure 1:**
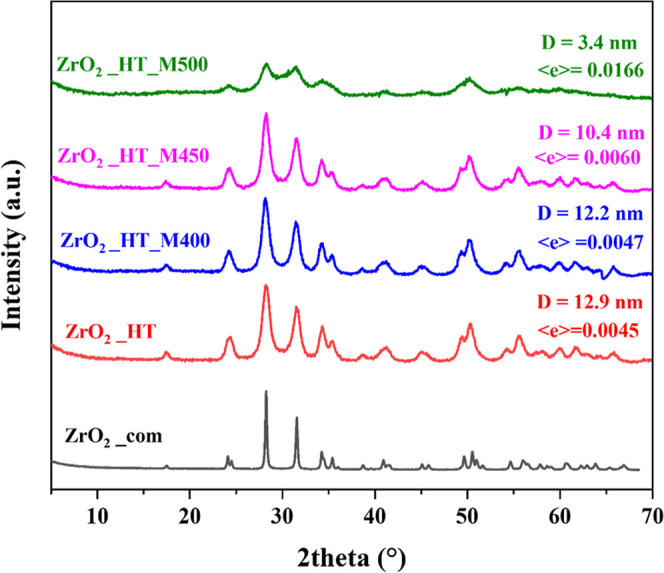
XRD patterns of hydrothermally treated and postmilled zirconia supports and commercial zirconia for comparison.

The full-profile Rietveld refinement (see Table S1) showed that the crystallite size and microstrain concentration barely change after postmilling at 400 rpm. However, an increase in milling intensity to 450–500 rpm leads to a significant decrease in the crystallite size from 10.4 to 3.4 nm, and the accumulation of microstrain in the crystal structure, represented by an increase in < *e* > value (see Figure 1). Sharp changes in these parameters are observed in the ZrO_2__HT_M500 sample, for which the diffraction pattern shows a strong background indicating partial amorphization. The lattice parameters of all postmilled samples also change significantly compared to the hydrothermal sample ZrO_2__HT.

#### 3.1.2 PDF Characterization of the Support

Next, the pair distribution function (PDF), a well-known technique for studying the local structure and defects in crystalline materials, was applied to obtain information about structural long- and short-range ordering in zirconia.^33^ Detailed and precise knowledge of atomic ordering is very important for a better understanding of the properties of the material, including surface characteristics, which are critical for heterogeneous catalytic processes. The data obtained from the PDF analysis complements the X-ray diffraction results, thanks to a much higher sensitivity to the local ordering of materials. Although the structure of different ZrO_2_ phases has been studied using this method in several works,^34–36^ the effect of milling on the local structural distortions of ZrO_2_ is poorly covered in the literature.^23^

The PDF of highly crystalline commercial ZrO_2_, shown in Figure 2, in general, coincides with that described earlier.^24^ The peak at the shortest *r* value can be assigned to Zr–O bond distances. The next two strongest peaks correspond to the nearest-neighbor Zr–Zr distances. The first of them represents Zr–Zr, whose polyhedra share an edge through two bridging O atoms, while the second peak is due to Zr–Zr pairs whose polyhedra share corners through a single bridging O atom, and are farther apart. For all support samples, the first peak at 2.10 Å corresponds to the first neighbor Zr–O, the second peak at 3.50 Å, and the third peak at 3.99 Å to the first and second neighbor Zr–Zr distances, respectively, as shown in Figure 2 inset, which are very close to the peaks associated with the monoclinic phase.^37,38^ The positions of the low-*r* peaks (at distances shorter than 5 Å, i.e., within the unit cell) in the experimental PDF analysis shown in Figure 2 exhibit virtually no changes with milling intensity (samples ZrO_2__HT_M400, ZrO_2__HT_M450, ZrO_2__HT_M500). As a result, the first peak (Zr–O) for all samples is identical, then the higher-*r* peaks exhibit various intensities since the samples have different degrees of long-range order. The intensities of the peaks for hydrothermal ZrO_2__HT and postmilled samples (ZrO_2__HT_M400, ZrO_2__HT_M450, and ZrO_2__HT_M450) fall off more quickly compared to commercial ZrO_2_, so these decreases are due to actual loss of structural correlation at long distances. Thus, distinct features disappear already at 30–50 Å, depending on the intensity of milling, while for commercial ZrO_2_ it is observed only after 70 Å, see Figure S1 of the Supporting Information.

**Figure 2:**
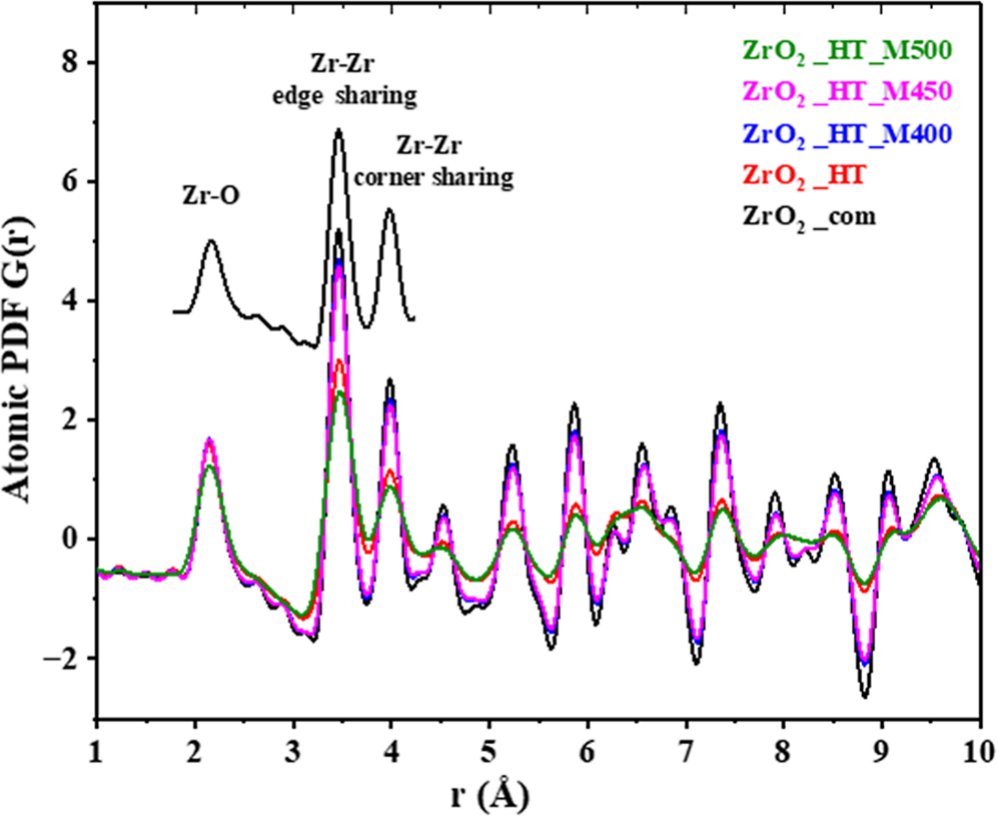
Atomic PDF for prepared ZrO_2_ samples in the low-*r* range. The zoomed graph (inset) includes the PDF of commercial ZrO_2_ with peak description.

The ZrO_2__HT_M500 sample is different from the others. Not only does it have the widest and least intense peaks that fall the fastest, but its first peak is also wider than the rest. This means the ZrO_7_ polyhedra are more distorted and irregular in the framework of this sample, giving a wider distribution of Zr–O distances. PDF analysis usually cannot reveal defects unless they are of high concentration (several percent at least). Still, the broader first peak of the ZrO_2__HT_M500 sample does seem to indirectly indicate a more disordered local environment, even at the shortest length scales, which is consistent with the Rietveld refinement. As seen in Figure 2, the features of the ZrO_2__HT_M500 sample slightly resemble ZrO_2__HT in intensity and development of the features, but this comes from a very different origin.

#### 3.1.3 SEM and Porosity Characterization of the Support

SEM micrographs, gathered in Figure S2 of the Supporting Information, confirm the initial diminution of the zirconia particles with milling, followed by agglomeration under the most intensive milling conditions, visible for sample ZrO_2__HT_M500.

The isotherms of nitrogen adsorption–desorption of the obtained samples are presented in Figure S3a of the Supporting Information indicates that all have a developed porous structure, as indicated by the presence of well-pronounced capillary-condensation loops, confirming their mesoporous characteristics.

The commercial zirconia sample (ZrO_2__com) isotherm is close to type I according to the IUPAC classification,^39^ however, it also contains a weakly pronounced capillary-condensation hysteresis. This indicates that ZrO_2__com has a mixed micromesoporous structure, which is confirmed by the calculation using t-plot and BJH method: volume of micro- (*V*_mi_) and mesopores (*V*_me_) is 0.03 and 0.04 cm^3^/g, respectively. Their sum equals the total pore volume *V* (0.07 cm^3^/g), determined at a relative nitrogen vapor pressure close to 1. Due to micropore fracture, this sample has the largest specific surface area (*S*) – 130 m^2^/g. This correlated with the mesopore size distribution (PSD) curve for the commercial sample, as it has a weakly expressed maximum close to the microporosity region, at 2.8 nm (Figure S3b).

Compared to the commercial ZrO_2_, isotherms of a different shape were obtained for hydrothermal and postmilled samples. ZrO_2__HT has a uniform mesoporous structure with a smaller specific surface area, but a larger volume and diameter of mesopores (Table 2). The latter was also indicated by the PSD curves shown in Figure S3b, based on which the diameter of the mesopores (*d*_me_) was established. In general, postmilling of the ZrO_2__HT sample leads to certain destruction of the porous structure and a corresponding decrease in the specific surface area and mesopore volume (Table 2). The mesopore volume (*V*_me_) for all modified samples coincides with the total pore volume *V*, which indicates the absence of mesopores in their structure.

**Table 2:** Parameters of the Porous Structure of the Initial and Modified Samples^a^

sample	SSA [m^2^/g]	*V*_mi_ [cm^3^/g]	*V*_me_ [cm^3^/g]	*d*_me_ [nm]
ZrO_2__HT	66	-	0.19	7.8
ZrO_2__HT_M400	68	-	0.14	6.6
ZrO_2__HT_M450	72	-	0.17	3.6; 5.8
ZrO_2__HT_M500	52	-	0.11	3.6; 32
ZrO_2__com	130	0.03	0.04	2.8

a (SSA) specific surface area, (*V*_mi_) volume of micropores, (*V*_me_) mesopore volume, (*d*_me_)-diameter of the mesopores.

The mesopore diameter also decreases after milling, particularly for samples ZrO_2__HT_M450 and ZrO_2__HT_M500, but their PSD curves have some common features. Thus, there is a significant shift of the main maximum toward a smaller pore diameter compared to the hydrothermal sample and the sample milled at 400 rpm (ZrO_2__HT_M400). In addition, the second diffused maximum is present in the region of larger diameter (this maximum is located beyond the presented range of pore sizes for sample ZrO_2__HT_M500). Consequently, these two samples have a bimodal mesoporous structure with approximately the same contribution of pores of both sizes to the total mesopore volume. All the trends described here are typical for dry milling of oxides with a high specific surface area.^25,40^ Overall, the samples derived from precipitated ZrO_2_, whether hydrothermally treated or milled, exhibit mesopores that are substantially larger than those found in the commercial ZrO_2__com sample. Among them, ZrO_2__HT and ZrO_2__HT_M400 stand out with the largest mesopores, offering the most extensive and accessible surface area for interaction with reactant molecules (Table 2). At the same time, the ZrO_2__HT_M450 sample may have a lower accessible surface area due to the smaller mesopore size and their distribution, in particular, a significant fraction of mesopores with a diameter of less than 4 nm (Figure S3b). For the ZrO_2__HT_M500 sample, a noticeable decrease in the specific surface area and mesopore volume caused by the partial destruction of the ZrO_2_ framework is observed.

#### 3.1.4 ATR-FTIR and Raman Characterization of the Support

The spectra from attenuated total reflectance Fourier transform infrared spectroscopy (ATR-FTIR) and Raman spectroscopy were acquired for initial characterization of the bare zirconia supports and are presented in Figures S4 and S5 of the Supporting Information, respectively.

Both types of spectra indicate that all samples correspond to the monoclinic zirconia structure; however, milling induces a rearrangement of the surface crystal lattice and extended milling results in partial structural degradation. In addition, the presence of residual nitrates was detected, likely remaining in the material due to the relatively low calcination temperature.

### 3.2 Catalytic Performance of Ni/ZrO_2_ and Structure

Catalytic performance of ZrO_2_ supports and Ni/ZrO_2_ assemblies.

First, the bare zirconia supports (ZrO_2__HT, ZrO_2__HT_M400, ZrO_2__HT_M450, ZrO_2__HT_M500) and the commercial ZrO_2_ were tested, with results summarized in Table S3 of the Supporting Information. Commercial ZrO_2_ was not active, while the synthesized supports showed very limited activity, with CH_4_ conversion below ∼10% and CO_2_ conversion within the error margin.

Next, the corresponding catalysts with nickel were investigated under identical conditions for their performance in dry methane reforming, with the results shown in Table S3. After reduction, the Ni/ZrO_2_ catalysts showed low conversions for both reactants at 550 °C. After increasing the temperature to 600 °C, the Ni/ZrO_2__HT_M400 catalyst, i.e. modified with mild milling intensity, showed the highest activity with ca. 29 and 39% conversion for CH_4_ and CO_2_ respectively, resulting in a yield of 22% for H_2_ and 32% for CO. Conversely, the catalyst without milling (Ni/ZrO_2__HT) and those with more intensive milling (Ni/ZrO_2__HT_M450 and Ni/ZrO_2__HT_M500) showed a significantly lower activity at the same temperature. The increase in temperature to 650 °C led to a temporary increase in activity of the catalyst without milling (Ni/ZrO_2__HT), improved to 17 and 23% conversion for CH_4_ and CO_2_, respectively, reaching similar activity as ZrO_2__HT_M400 with 17 and 25% CH_4_ and CO_2_ conversion, respectively. The catalysts with more intensive milling of the supports (Ni/ZrO_2__HT_M450 and Ni/ZrO_2__HT_M500) were not affected by the temperature increase to 650 °C and exhibited negligible activity, achieving conversions of less than 8% for both reactants. For the results of conversion as a function of the time on stream, see Figure S6 of the Supporting Information, and for a comparison between the current study and previous reports on similar catalytic systems, see Table S4 of the Supporting Information.

The catalytic results show that activity increased for the mildly milled support, highlighting the potential of milling as a tool for tuning catalytic performance. Moreover, in the active samples, CO_2_ conversion exceeded that of CH_4_, which may indicate enhanced CO_2_ activation on these modified catalysts.

#### 3.2.1 *Operando* Raman Characterization of the Catalyst

To gain information about the catalyst structure under reaction conditions, *operando* Raman spectroscopy was employed for characterization of the active Ni–ZrO_2_ assemblies (Ni/ZrO_2__HT, Ni/ZrO_2__HT_M400, and Ni/ZrO_2__HT_M450) from the reduction step to the reaction (CH_4_:CO_2_ 1:1). For insights on the spectroscopic benchmarks, see Table S5 in the Supporting Information. Before reduction, the presence of NiO is shown by the broad feature in the Raman spectra around 500 cm^–1^ and possibly above 1000 cm^–1^ (Figure 3).^41^ As expected, these bands vanish after reduction. The disappearance of the band at ca. 1000 cm^–1^ also shows that the residual nitrates, which remained after the synthesis, are finally degraded in the activation step at high temperature before reaction. The spectra of Ni/ZrO_2__HT (Figure 3a) acquired at RT exhibit a shoulder at 90 cm^–1^, a sharp Raman shift at 181 cm^–1^, less sharp at 336 and 381 cm^–1^, the largest and broadest shift at 256 cm^–1^, then 610 cm^–1^ and at 424 cm^–1^, which are typically associated with monoclinic ZrO_2_.^42^ During the reduction at 550 °C, additional doublets at 211 and 230 cm^–1^, and at 523 and 545 cm^–1^ appear, which are also characteristic of monoclinic ZrO_2_. Under interaction with the feedstock, CO_2,_ and CH_4_ at 550 °C, the shifts at 139 and 260 cm^–1^ attributed to the tetragonal phase start to appear. At 650 °C, some of the typical features of monoclinic ZrO_2_ can still be recognized, mainly at 174, 321, 465, and 620 cm^–1^. After cooling down to room temperature, the typical signals of monoclinic ZrO_2_ are well recognized, together with weak modes at 139 and 252 cm^–1^, characteristic of tetragonal ZrO_2_ (see Table S2 in the Supporting Information). The shift of the second signal from 260 cm^–1^ to 252 cm^–1^ could be ascribed to the increase in the number of oxygen vacancies.^43^ The latter can form due to the entry of Ni^2+^ cations into the structure at high temperatures (650 °C), which will be discussed below.

**Figure 3:**
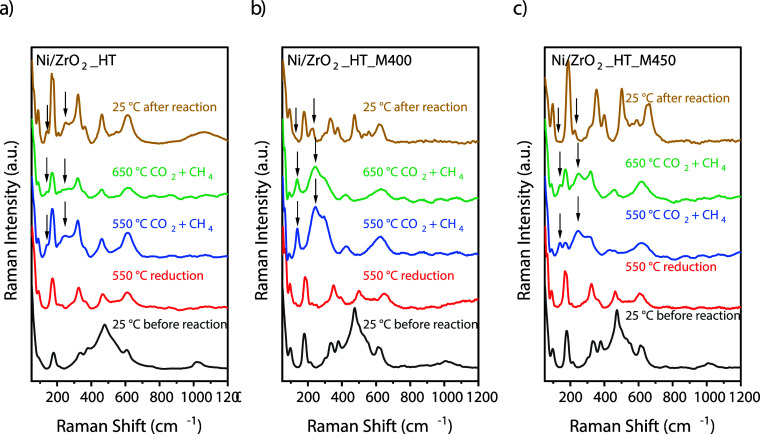
Raman spectra of the Ni–ZrO_2_ assemblies prepared by the intimate mixing of 10% wt. NiO with (a) ZrO_2__HT, (b) ZrO_2__HT_M400, and (c) ZrO_2__HT_M450 under dry methane reforming reaction conditions.

Sample Ni/ZrO_2__HT_M400 presented in Figure 3b shows similar features to Ni/ZrO_2__HT at RT conditions. During the reduction at 550 °C, Raman shifts typical for monoclinic ZrO_2_ were also observed. However, following the switch to the reaction mixture, the emergence of strong intensity modes at 139 and 260 cm^–1^ occurred at the expense of the band at 181 cm^–1^, indicating the presence of the tetragonal phase. These modes are the most intense after exposure to the reaction mixture, which may indicate the formation of a significant fraction of the tetragonal phase under these conditions. Even so, after increasing the temperature to 650 °C, the intensity of the latter peaks decreased while shoulders reappeared at the positions of monoclinic ZrO_2_. Upon subsequent cooling to RT, the tetragonal phase-linked signals remain detectable. While the bands associated with monoclinic ZrO_2_ become more prominent, spectra indicate that the sample still contains a mixture of tetragonal and monoclinic phases, with prevailing monoclinic content.

Sample ZrO_2__HT_M450 (Figure 3c) displayed a similar band broadening at 25 °C at room temperature, and Raman modes were observed at 90, 174, 211, 321, 370, 461, 620, with weaker signals at 300, 523, and 546 cm^–1^, indicative of a predominant monoclinic phase. Exposure to the reaction mixture at 550 °C also led to the emergence of bands at 139 and 250 cm^–1^, revealing the appearance of the tetragonal phase alongside the monoclinic phase. At 650 °C, bands corresponding to the monoclinic phase intensified more than on the Ni/ZrO_2__HT_M400 sample. Upon cooling to room temperature, the tetragonal phase modes finally nearly disappear. Compared with the milder-milled ZrO_2__HT_M400 sample, the monoclinic phase is more prominent in ZrO_2__HT_M450 after reaction, although tetragonal contributions are still detectable. Rietveld refinement reveals a minor contribution (2%) of the tetragonal phase, with monoclinic ZrO_2_ and metallic Ni comprising 92% and 6% respectively, of the sample ZrO_2__HT_M450 after reaction. For the XRD pattern of the spent ZrO_2__HT_M450, see Figure S7 in the Supporting Information. The average crystallite size of the monoclinic phase was calculated to be 8.5 nm.

Operando Raman spectroscopy provides important insight into the structural changes occurring under reaction conditions. The results indicate that these changes are linked to milling-induced lattice deformation, which facilitates the transition from monoclinic to tetragonal zirconia. Interestingly, this transformation occurs only in the presence of Ni, suggesting an enhanced interaction between the active phase and the support. Based on these results, it can be assumed that the largest fraction of the tetragonal phase is formed for sample Ni/ZrO_2__HT_M400, and the smallest fraction for sample Ni/ZrO_2__HT_M450.

### 3.3 Nature of Milled-Induced Defects in the Bare ZrO_2_ Supports

Since many types of defects, including paramagnetic ones, are present in milled oxides, electron paramagnetic resonance (EPR) spectroscopy is one of the most suitable methods for studying such materials. Several papers have described paramagnetic defects in ZrO_2_ material due to calcination temperatures, irradiation for photocatalytic applications, or absorption of gases caused by electron transfer in the solid material.^44–47^ The major paramagnetic defects in ZrO_2_ described in the literature are attributed to several Zr^3+^ reduced sites in the solid and on the surface. The others are attributed to trapped single electrons located in oxygen vacancies of ZrO_2_ (signal centered at *g* = 2.002) and electrons transferred to absorbed acceptors (O_2_), molecular oxygen radical anions O_2_^–^ species.

EPR spectra of the bare zirconia supports were acquired to establish the nature of the defects induced by milling. The spectrum of the ZrO_2__HT sample acquired before reaction contains signals at *g* = 2.062 and 1.972, see Figure 4a, assigned to O_2_^–^ species and Zr^3+^ ions presence, respectively.

**Figure 4:**
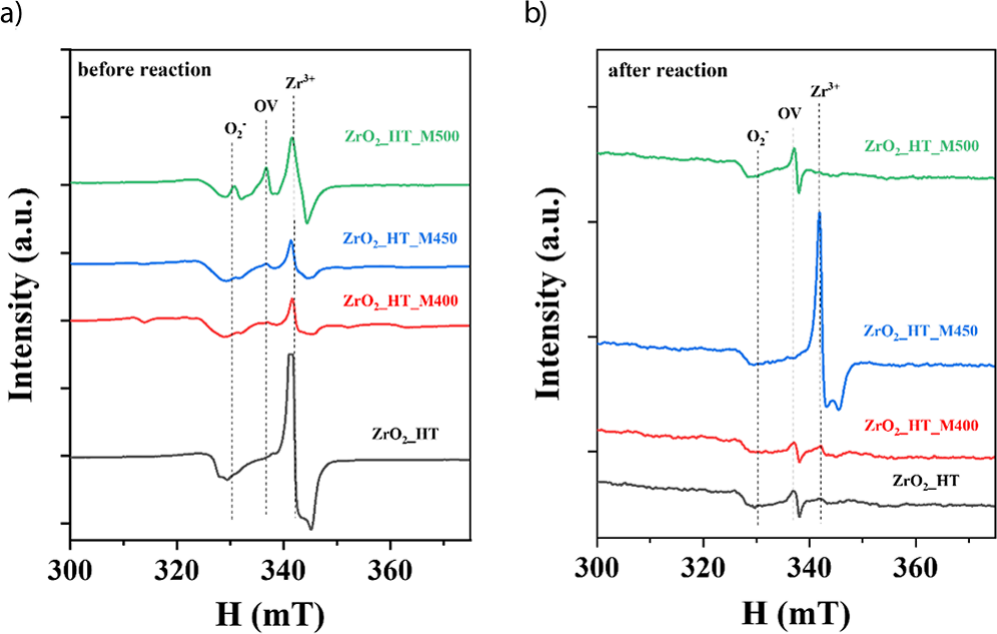
EPR spectra recorded at −160 °C for all support materials: (a) before and (b) after exposure to reaction conditions.

Subsequent milling of this sample at 400 rpm (sample ZrO_2__HT_M400) results in a sharp decrease in the intensity of the signals associated with Zr^3+^ defects (*g* = 1.972). Milling at 450 rpm slightly reduces the signal, whereas increasing the speed to 500 rpm results in a significant growth in intensity. In addition, new signals appear in the spectra of the milled samples. These include a signal at *g* = 2.002 associated with unpaired electrons located in oxygen vacancies, so-called “trapped electrons”.^45,46,48^ The intensity of this signal rises with the milling intensity. It is generally accepted that the higher the intensity of this signal (2.001–2.003), the higher the concentration of oxygen vacancies.^21,49–51^ At the same time, the intensity of the signal with *g* = 2.062 (O_2_^–^ species) hardly changes after milling at different intensities.

Significant changes are observed in the spectra of the samples after reduction and subsequent exposure to the reaction mixture under heating and cooling, as shown in Figure 4b. It should be noted that the EPR spectra before and after the reaction were measured using different amounts of material (see details in Experimental section); therefore, the intensity of the EPR signals before and after the reaction cannot be compared, unlike the positions of the signals represented by the *g*-factor. The intensity of the signal at *g* = 2.062 (O_2_^–^ species) remains practically unchanged in all samples, yet the other two signals show different trends. The signal associated with unpaired electrons located in oxygen vacancies becomes the most intense after reaction for most samples. An exception is ZrO_2__HT_M450, where the dominant contribution arises from Zr^3+^ species. These differences may be related to changes in phase composition occurring during the reduction and heating–cooling cycles in the reaction mixture. It is known that the distribution of electrons between oxygen vacancies and Zr^3+^ ions is not necessarily 1:1.^45,48^ Therefore, the pronounced increase in the Zr^3+^ signal intensity accompanied by a decrease in the oxygen vacancy signal for the ZrO_2__HT_M450 sample may reflect electron redistribution under high-temperature reaction conditions. This apparent imbalance can be rationalized by the interaction of oxygen vacancies with neighboring Zr^4+^ ions, leading to their partial reduction to Zr^3+^.^45,52^

The above results confirm the introduction of Zr^3+^ oxygen vacancies and O_2_^–^ species defects into the milled structure, among which Zr^3+^ and oxygen vacancies are partially healed during DMR reaction.

## 4 Discussion

### 4.1 Stability of the Defects in Zirconia

The key role of oxygen vacancies in the DMR process is associated with enhanced CO_2_ adsorption due to the lowering of the energy barrier,^13^ followed by the formation of labile oxygen species able to oxidize the carbon deposit.^9,14–16^ The latter enables maintaining the catalytic activity of the material; thus, it is highly desirable.

As shown with the presented data from PDF analysis and the results of the Rietveld refinement, the mildly milled zirconia support samples (ZrO_2__HT_M400 and ZrO_2__HT_M450) are single-phase monoclinic materials that have inherited local atomic ordering within the first coordination shell, extending to a distance of less than 10 nm from the hydrothermal monoclinic sample ZrO_2__HT. Further increasing the milling intensity (sample ZrO_2__HT_M500) results in an increase in structural distortions, which reduces the length of structural coherence to several nanometers and causes a change in the type of long-range order, while maintaining the local atomic structure relatively intact. The EPR spectra confirmed the presence of Zr^3+^, oxygen vacancies, and O_2_^–^ defects in the structure of the zirconia support materials, the concentration of which depends on the intensity of milling, obtaining the highest content of oxygen vacancies for the most intensively milled sample (ZrO_2__HT_M500) and their lowest content for sample ZrO_2__HT_M450, according to EPR data. To investigate the thermal stability of the introduced defects, bare support samples were subjected to a temperature ramp under inert gas flow in the diffuse reflectance cell. The acquired diffuse reflectance spectra (DRIFTS) in the far-infrared region exhibit the same spectroscopic features before and after the experiment (see Figure S8 of the Supporting Information). In the spectra, one can identify the characteristic band positions of monoclinic ZrO_2_ ^53,54^ and low intensity bands of tetragonal ZrO_2_ ^55^ hindering clear phase differentiation. However, the reversibility of the features at 25 °C before and after the procedure suggests stability of the material’s structure under those conditions. Moreover, the EPR spectra recorded for zirconia support materials (without nickel) subjected to reaction conditions indicate the stability of the introduced Zr^3+^ defects for sample ZrO_2__HT_M450, while for other samples, they are no longer detected. Oxygen vacancies’ contribution is the highest for the most milled sample but is relatively high for all samples in comparison to their spectra before the reaction. This suggests that during the reaction conditions, zirconia supports undergo partial “healing” of the structure, which is not related to oxygen vacancies, and therefore maintain their stable, high potential for CO_2_ activation.

For the Ni/ZrO_2_ assemblies, an additional route of defect occurrence should be considered, connected with the replacement of the Zr^4+^ cations in the ZrO_2_ lattice with Ni during the elevated temperatures of the reaction. As shown in the works,^13,14,56^ this phenomenon is more probable in the presence of a tetragonal rather than a monoclinic zirconia phase, due to the similarity of the former lattice parameters with those of NiO. Although tetragonal ZrO_2_ is considered less suitable as a support because its inevitable transformation into the monoclinic phase at higher temperatures limits the activity of Ni/oxide catalysts in the DMR process,^17^ the DFT calculations reported in the literature show that the energy barrier of CO_2_ dissociation on the surface of tetragonal ZrO_2_ is lower compared to that for the monoclinic phase.^9^ Therefore, the presence of a small fraction of the tetragonal phase in the structure of ZrO_2_ support, which can be stabilized by nickel, strengthening the interaction between nickel and zirconia, improves the catalytic performance, as previously reported.^16,57^ However, the low 2% content of the tetragonal phase for sample Ni/ZrO_2__HT_M450 is obviously insufficient, since this sample did not show activity. Moreover, as mentioned above, this milled support contains the smallest amount of oxygen vacancies.

In the case of NiO deposited on the surface of a milled ZrO_2_ by means of intimate, but mild mixing at room temperature, their contact is rather loose, but Ni can be primarily anchored at oxygen vacancies without forming strong Ni–O–Zr bonds.^58^ The following pretreatment in hydrogen at 550 °C, aiming at Ni reduction, favors the interaction between nickel and zirconia, facilitating Ni introduction into the ZrO_2_ structure, causing the formation of additional defects, and inducing regional phase change to balance it. However, for the ZrO_2__HT sample, which contains almost no oxygen vacancies before the reaction, according to the EPR results (Figure 4a), part of the deposited Ni can be introduced into the ZrO_2_ lattice with the formation of Ni–O–Zr bonds and oxygen vacancies during subsequent heating in the reaction mixture. The H_2_-TPR profiles collected during the reduction of the Ni-zirconia samples (see Figure S9, Supporting Information) indicate the presence of two distinct Ni species, as evidenced by two clearly separated hydrogen consumption peaks. These reduction stages are attributed to more weakly interacting Ni species and to Ni incorporated into or strongly interacting with the zirconia lattice, in agreement with previous reports.^59–62^ For Ni/ZrO_2__HT, the reduction peaks appear at lower temperatures (223 and 269 °C) than for catalysts supported on milled zirconia, where they are observed at 231 and 314 °C for ZrO_2__HT_M400 and 232 and 310 °C for ZrO_2__HT_M450. The shift of the second reduction peak toward higher temperatures for the milled supports indicates a stronger Ni–ZrO_2_ interaction. This effect can be attributed to the higher concentration of oxygen vacancies generated during milling, which serve as preferential anchoring sites for nickel species. Thus, in catalysts based on milled supports, the Ni-support interaction is largely governed by the presence of oxygen vacancies.

The strengthened metal–support interaction enhances the resistance of Ni particles to sintering, thereby mitigating one of the primary causes of catalyst deactivation in the DMR reaction. Consistently, Raman spectroscopy reveals the formation of tetragonal ZrO_2_ under reaction conditions in Ni/ZrO_2_ systems, with partial retention of this phase upon cooling to room temperature. This stabilization effect is most pronounced for catalysts supported on mildly milled zirconia.

### 4.2 Structure–activity Relationship

Our results show that milling of the zirconia support influences the performance of the Ni/ZrO_2_ catalyst, see data gathered in Table 3 and Figure S6 of the Supporting Information. The highest activity was reached for the catalyst with mildly milled zirconia support (Ni/ZrO_2__HT_M400). It results from the/i/maximum preservation of the crystalline and porous structure, especially large mesopore size and the specific surface area;/ii/introduction of a sufficient (optimal) number of oxygen vacancies into the structure;/iii/creation of prerequisites for the formation of a fraction of tetragonal ZrO_2_ after the deposition of NiO and pretreatment in hydrogen and reaction mixture. It is noteworthy that the effect of milling intensity was partially compensated for the catalyst without milling by increasing the temperature. As shown in Table 3 for Ni/ZrO_2__HT, the activity increased when the temperature rose to 650 °C and became closer to that of Ni/ZrO_2__HT_M400, the most active catalyst at the same temperature. This can be explained by the formation of a certain number of oxygen vacancies (but not the optimal one) at the stage of reduction of this catalyst at 550 °C, as shown previously for ZrO_2_.^22,43^ Oxygen vacancies are key basic defect sites in reducible oxides that significantly enhance CO_2_ activation during dry methane reforming.^9^ Owing to its linear geometry and electron-deficient nature, CO_2_ preferentially adsorbs on electron-rich basic sites such as oxygen vacancies. Charge transfer at these sites weakens the C=O bonds and promotes bending of the molecule, thereby lowering the barrier for activation and dissociation into CO and reactive oxygen species. The resulting oxygen species can participate in subsequent oxidation of carbonaceous intermediates, helping to mitigate coke formation and sustain catalytic performance under DMR conditions.^63^

**Table 3:** Performance of the Ni/ZrO_2_ Catalysts under dry Methane Reforming Conditions^a^

catalyst	temperature, °C	CH_4_ conversion, %	CO_2_ conversion, %	H_2_ yield, %	CO yield, %
Ni/ZrO_2__HT	550	7	5	1	2
	600	6	1	0	0
	650	17	23	9	16
Ni/ZrO_2__HT_M400	550	5	0	0	0
	600	29	39	22	32
	650	17	25	12	23
Ni/ZrO_2__HT_M450	550	6	1	0	0
	600	10	11	3	7
	650	5	1	1	2
Ni/ZrO_2__HT_M500	550	5	2	0	1
	600	0	0	0	0
	650	2	0	0	0

a *values are presented as an average over 1 h at the given temperature.

Oxygen vacancies may be additionally formed at 600–650 °C during the reaction due to the introduction of Ni into the tetragonal ZrO_2_ lattice, as mentioned above. This is also evident from the EPR spectra (Figure 4b). Nevertheless, Ni/ZrO_2_ assemblies with support milled at higher intensity (450 and 500 rpm) did not show enhanced activity with increasing temperature, disclosing that overmilling the support significantly alters the catalyst’s chemistry, preventing increased activity even with higher temperatures and despite high oxygen vacancy concentration. Besides, these samples have mesopores with a size of 3.6 nm (Table 2). At the same time, it is believed that the supports should have a pore size ≥5 nm for rapid mass transfer of reagents to active centers, and a lower ability of the pores to sinter and clog with coke deposit at higher temperatures.^64–66^ For sample ZrO_2__HT_M500, the lack of activity is most likely connected with an excessive concentration of oxygen vacancies and, as a result, the partial collapse of the crystal and porous structure.^5^ Therefore, low-intensity milling allows the introduction of an optimal amount of oxygen vacancies while maintaining the crystalline and porous structure. Still, for stabilizing tetragonal zirconia, the crucial factor is the presence of nickel.

It should be noted that the Ni/ZrO_2_ assemblies studied under DMR conditions do not maintain constant activity, due to rather loose contact between Ni and zirconia components (intimate mixing), but their performance profile still reveals meaningful trends. Based on these trends, it can be concluded that the support plays a decisive role in determining the catalytic performance, as the Ni component remains identical across all samples, originating from the same commercial material and simply mixed with each support, thus ruling out effects typically introduced during chemical deposition (e.g., changes in particle size or morphology). From the catalytic results, it is apparent that the conversion of CO_2_ is a few % higher for the active Ni/ZrO_2_ assemblies. This agrees with the well-established role of oxygen vacancies in promoting CO_2_ adsorption^16^ and is consistent with the EPR data (Figure 4a).

## 5 Conclusions

This work demonstrates that mechanical milling can be employed as an effective approach to tailor the defect structure of zirconia supports and thereby modulate the performance of Ni-based catalysts in dry methane reforming. Milling introduces three distinct types of defects into the ZrO_2_ lattice, whose nature, concentration, and thermal stability were systematically investigated, together with their evolution under reaction conditions. After milling, the formed stable monoclinic phase contains varying amounts of oxygen vacancies. These vacancies govern the degree of interaction between nickel and the support and influence the transformation of the monoclinic phase into the tetragonal one under reaction conditions. Among the studied materials, mildly milled zirconia obtained at 400 rpm produced the most active catalyst, reaching CH_4_ and CO_2_ conversions of 29% and 39% at 600 °C, respectively. This behavior is attributed to an optimal concentration of oxygen vacancies that enhances interaction between the zirconia support and Ni.

Upon reduction and during reaction, this enhanced metal–support interaction leads to mutual stabilization of the active phase and the support and is accompanied by the emergence of a tetragonal ZrO_2_ fraction. The phase transformation is therefore considered a consequence of milling-induced structural and defect-related modifications rather than the direct origin of the improved catalytic performance. These conclusions are supported by PDF analysis and EPR spectroscopy, which provide consistent evidence for the formation and stability of specific defect species in the postmilled zirconia.

Importantly, controlled defect engineering through milling is achieved without significant degradation of the textural properties of the support, enabling enhanced CO_2_ activation at oxygen vacancies while preserving catalyst stability. By establishing a direct relationship between milling-induced structural modifications of ZrO_2_ and the catalytic behavior of supported Ni catalysts, this study highlights support defect engineering as a viable and conceptually distinct strategy for catalyst optimization, with implications extending beyond dry methane reforming.

## Supplementary Material



## Notes

Supplied data on lattice parameters, full range atomic PDF spectra, micrographs, porosity analysis, together with details on catalytic testing and in situ/operando FTIR and Raman characterization (PDF)

## References

[1] Kauppi E. I., Honkala K., Krause A. O. I., Kanervo J. M., Lefferts L. (2016). ZrO2 Acting
as a Redox Catalyst. Top. Catal..

[2] Fatimah I., Yanti I., Suharto T. E., Sagadevan S. (2022). ZrO2-based
catalysts for biodiesel production: A review. Inorg. Chem. Commun..

[3] Rani V., Sharma A., Kumar A., Singh P., Thakur S., Singh A., Le Q. V., Nguyen V. H., Raizada P. (2022). ZrO2-based
photocatalysts for wastewater treatment: from novel modification strategies
to mechanistic insights. Catalysts.

[4] Lou Y., Steib M., Zhang Q., Tiefenbacher K., Horváth A., Jentys A., Liu Y., Lercher J. A. (2017). Design
of stable Ni/ZrO2 catalysts for dry reforming of methane. J. Catal..

[5] Fakeeha A., Kurdi A., Al-Baqmaa Y., Ibrahim A., Abasaeed A., Al-Fatesh A. (2022). Performance
Study of Methane Dry Reforming on Ni/ZrO2
Catalyst. Energies.

[6] Li K., Chen J. G. (2019). CO2 Hydrogenation to Methanol over ZrO2-Containing
Catalysts: Insights into ZrO2 Induced Synergy. ACS Catal..

[7] Akune T., Morita Y., Shirakawa S., Katagiri K., Inumaru K. (2018). ZrO­(2) Nanocrystals
As Catalyst for Synthesis of Dimethylcarbonate from Methanol and Carbon
Dioxide: Catalytic Activity and Elucidation of Active Sites. Langmuir.

[8] Jia X., Zhang X., Rui N., Hu X., Liu C.-j. (2019). Structural
effect of Ni/ZrO2 catalyst on CO2 methanation with enhanced activity. Appl. Catal., B.

[9] Niu J., Zhang C., Liu H., Jin Y., Zhang R. (2023). Enhanced performance
of oxygen vacancies on CO2 adsorption and activation over different
phases of ZrO2. Front. Energy.

[10] Ozkan D. M., Uzun A., Caglayan B. S., Aksoylu A. E. (2023). A DFT study on the
role of oxygen vacancy on m-ZrO2 (1̅11) in adsorption and dissociation
of CO2. Surf. Sci..

[11] Zhang M., Zhou X., Yang J., Yang T., Liu Z., Han Y. (2023). Deciphering the ZrO2 phase engineering effects on dry reforming of
methane over the Ni/ZrO2 catalysts. Fuel.

[12] Wang H., Li Q., Chen J., Chen J., Jia H. (2023). Efficient Solar-Driven
CO(2) Methanation and Hydrogen Storage Over Nickel Catalyst Derived
from Metal-Organic Frameworks with Rich Oxygen Vacancies. Adv. Sci. (Weinh).

[13] Du H., Shao Z. (2025). A Review on Modulating Oxygen Vacancy Defect of Catalysts to Promote
CO2 Reduction Reaction to CO. Energy Fuels.

[14] Li W., Zhao Z., Ding F., Guo X., Wang G. (2015). Syngas Production
via Steam–CO2 Dual Reforming of Methane over LA-Ni/ZrO2 Catalyst
Prepared by l-Arginine Ligand-Assisted Strategy: Enhanced Activity
and Stability. ACS Sustainable Chem. Eng..

[15] Wei M., Shi X. (2024). Research Progress on
Stability Control on Ni-Based Catalysts for
Methane Dry Reforming. Methane.

[16] Zhang M., Yang T., Jiang K., Gao Y., Yang J., Liu Z., Han Y. (2024). Rationally constructing
metastable ZrO2 supported Ni
catalysts for highly efficient and stable dry reforming of methane. Appl. Catal. B Environ.

[17] Al-Fatesh A. S., Bamatraf N. A., Alreshaidan S. B., Abu-Dahrieh J. K., patel N., Ibrahim A. A., Fakeeha A. H., Jumah A. b., Kumar R. (2024). Cost-Effective Single-Step Synthesis
of Metal Oxide-Supported Ni
Catalyst for H2-Production Through Dry Reforming of Methane. Arabian J. Sci. Eng..

[18] Sydorchuk V., Levytska S., Kiziun O., Vasylechko L., Simkovicova K., Valtera S., Billinghurts B. E., Vajda S., Olszowka J. E. (2025). Combined hydrothermal and mechanochemical
control of structural modifications of zirconium dioxide for catalytic
applications. RSC Mechanochem..

[19] Shahzadi A., Iqbal M. A., Majeed A., Yasmeen I., Akmal M., Ejaz S., Fatima S., Arshad M. N., Asad M. (2026). Progress in
mechanochemical synthesis of catalysts for the CO 2 processes: a step
towards carbon neutrality. RSC Adv..

[20] Zhan F., Wen G., Li R., Feng C., Liu Y., Liu Y., Zhu M., Zheng Y., Zhao Y., La P. (2024). A comprehensive review
of oxygen vacancy modified photocatalysts: synthesis, characterization,
and applications. Phys. Chem. Chem. Phys..

[21] Niu K., Liu Q., Liu C., Yu Z., Zheng Y., Su Y., Zhao Y., Liu B., Cui S., Zang G. (2024). Unraveling the role of oxygen vacancies in
metal oxides: Recent progress
and perspectives in NH3-SCR for NOx removal. Chem. Eng. J..

[22] Eder D., Kramer R. (2002). The stoichiometry of
hydrogen reduced zirconia and
its influence on catalytic activity - Part 1: Volumetric and conductivity
studies. Phys. Chem. Chem. Phys..

[23] Gateshki M., Petkov V., Williams G., Pradhan S., Ren Y. (2005). Atomic-scale
structure of nanocrystalline ZrO 2 prepared by high-energy ball milling. Phys. Rev. B:Condens. Matter Mater. Phys..

[24] Gateshki M., Petkov V., Hyeon T., Joo J., Niederberger M., Ren Y. (2006). Interplay between the local structural
disorder and the length of
structural coherence in stabilizing the cubic phase in nanocrystalline
ZrO2. Solid State Commun..

[25] Kucio K., Charmas B., Sydorchuk V., Khalameida S., Khyzhun O. (2020). Synthesis and modification of Ce-Zr
oxide compositions
as photocatalysts. Appl. Catal., A.

[26] Huo F., Shen Y.-A., He S., Zhang K., Nishikawa H. (2021). Fabrication
of NiO/ZrO2 nanocomposites using ball milling-pyrolysis method. Vacuum.

[27] Steib M., Lou Y., Jentys A., Lercher J. A. (2017). Enhanced Activity in Methane Dry
Reforming by Carbon Dioxide Induced Metal-Oxide Interface Restructuring
of Nickel/Zirconia. ChemCatChem.

[28] Akselrud L., Grin Y. (2014). WinCSD: software package
for crystallographic calculations (Version
4). J. Appl. Crystallogr..

[29] Garvie R. C., Nicholson P. S. (1972). Phase Analysis
in Zirconia Systems. J. Am. Ceram. Soc..

[30] Toby B. H., Von Dreele R. B. (2013). GSAS-II:
the genesis of a modern open-source all purpose
crystallography software package. Appl. Crystallogr..

[31] Kadam S. A., Sandoval S., Bastl Z., Simkovicova K., Kvítek L., Jasik J., Olszowka J. E., Valtera S., Vaidulych M., Morávková J. (2023). Cyclohexane
oxidative dehydrogenation on graphene-oxide-supported cobalt ferrite
nanohybrids: effect of dynamic nature of active sites on reaction
selectivity. ACS Catal..

[32] Park J.-H., Yeo S., Chang T.-S. (2018). Effect of supports
on the performance of Co-based catalysts
in methane dry reforming. J. CO2 Util..

[33] Billinge S. J. (2008). Nanoscale
structural order from the atomic pair distribution function (PDF):
There’s plenty of room in the middle. J. Solid State Chem..

[34] Zhang F., Chupas P. J., Lui S. L. A., Hanson J. C., Caliebe W. A., Lee P. L., Chan S.-W. (2007). In situ study of
the crystallization
from amorphous to cubic zirconium oxide: Rietveld and reverse Monte
Carlo analyses. Chem. Mater..

[35] Wu C., Lin L., Liu J., Zhang J., Zhang F., Zhou T., Rui N., Yao S., Deng Y., Yang F. (2020). Inverse
ZrO2/Cu as a highly efficient methanol synthesis catalyst from CO2
hydrogenation. Nat. Commun..

[36] Pokratath R., Lermusiaux L., Checchia S., Mathew J. P., Cooper S. R., Mathiesen J. K., Landaburu G., Banerjee S., Tao S., Reichholf N. (2023). An amorphous phase precedes crystallization:
unraveling the colloidal synthesis of zirconium oxide nanocrystals. ACS Nano.

[37] Soo Y., Chen P., Huang S., Shiu T., Tsai T., Chow Y., Lin Y., Weng S., Chang S., Wang G. (2008). Local structures surrounding Zr in nanostructurally
stabilized cubic zirconia: structural origin of phase stability. J. Appl. Phys..

[38] Rijckaert H., De Roo J., Van Zele M., Banerjee S., Huhtinen H., Paturi P., Bennewitz J., Billinge S. J., Bäcker M., De Buysser K. (2018). Pair distribution function analysis of ZrO2
nanocrystals and insights in the formation of ZrO2-YBa2Cu3O7 nanocomposites. Materials.

[39] Thommes M., Kaneko K., Neimark A. V., Olivier J. P., Rodriguez-Reinoso F., Rouquerol J., Sing K. S. W. (2015). Physisorption of gases, with special
reference to the evaluation of surface area and pore size distribution
(IUPAC Technical Report). Pure Appl. Chem..

[40] Sydorchuk V., Khalameida S., Zazhigalov V., Skubiszewska-Zięba J., Leboda R., Wieczorek-Ciurowa K. (2010). Influence of mechanochemical activation
in various media on structure of porous and non-porous silicas. Appl. Surf. Sci..

[41] Anandha
Babu G., Ravi G., Hayakawa Y. (2015). Microwave synthesis and effect of
CTAB on ferromagnetic properties of NiO, Co3O4 and NiCo2O4 nanostructures. Appl. Phys. A: Mater. Sci. Process..

[42] Keramidas V. G., White W. B. (1974). Raman Scattering
Study of the Crystallization and Phase
Transformations of ZrO2. J. Am. Ceram. Soc..

[43] Zhao L., Zhao J., Wu T., Zhao M., Yan W., Zhang Y., Li H., Wang Y., Xiao T., Zhao Y. (2019). Synergistic Effect
of Oxygen Vacancies and Ni Species on Tuning Selectivity
of Ni/ZrO(2) Catalyst for Hydrogenation of Maleic Anhydride into Succinic
Anhydride and gamma-Butyrolacetone. Nanomaterials
(Basel).

[44] Matta J., Lamonier J.-F., Abi-Aad E., Zhilinskaya E. A., Aboukaïs A. (1999). Transformation of tetragonal zirconia
phase to monoclinic
phase in the presence of Fe 3+ ions as probes: an EPR study. Phys. Chem. Chem. Phys..

[45] Gionco C., Paganini M. C., Giamello E., Burgess R., Di Valentin C., Pacchioni G. (2013). Paramagnetic defects in polycrystalline zirconia: an
EPR and DFT study. Chem. Mater..

[46] Zhang J., Gao Y., Jia X., Wang J., Chen Z., Xu Y. (2018). Oxygen vacancy-rich
mesoporous ZrO2 with remarkably enhanced visible-light photocatalytic
performance. Sol. Energy Mater. Sol. Cells.

[47] Polliotto V., Livraghi S., Giamello E. (2018). Electron magnetic
resonance as a
tool to monitor charge separation and reactivity in photocatalytic
materials. Res. Chem. Intermed..

[48] Gorban O., Synyakina S., Volkova G., Gorban S., Konstantiova T., Lyubchik S. (2015). Formation of metastable tetragonal zirconia nanoparticles:
Competitive influence of the dopants and surface state. J. Solid State Chem..

[49] Chen L., Wang X., Cong Q., Ma H., Li S., Li W. (2019). Design of a hierarchical Fe-ZSM-5@
CeO2 catalyst and the enhanced
performances for the selective catalytic reduction of NO with NH3. Chem. Eng. J..

[50] Zhao W., Zhang K., Wu L., Wang Q., Shang D., Zhong Q. (2021). Ti3+ doped V2O5/TiO2
catalyst for efficient selective catalytic reduction
of NOx with NH3. J. Colloid Interface Sci..

[51] Rinaudo M. G., Beltrán A. M., Fernández M. A., Cadús L. E., Morales M. R. (2020). Tailoring materials
by high-energy ball milling: TiO2
mixtures for catalyst support application. Mater.
Today Chem..

[52] Benavides-Guerrero J.
A., Gerlein L. F., Angel-Ospina A. C., Fourmont P., Bhattacharya A., Zirakjou A., Vaussenat F., Ross C. A., Cloutier S. G. (2025). Room-temperature
laser crystallization of oxygen vacancy-engineered zirconia for additive
manufacturing. Addit. Manuf..

[53] Maczka M., Lutz E. T. G., Verbeek H. J., Oskam K., Meijerink A., Hanuza J., Stuivinga M. (1999). Spectroscopic studies of dynamically
compacted monoclinic ZrO_2_. J. Phys.
Chem. Solids.

[54] Zhang H., Liu Y., Zhu K., Siu G., Xiong Y., Xiong C. (1999). Infrared spectra
of nanometre granular zirconia. J. Phys.: Condens.
Matter.

[55] El
Boutaybi A., Cervasio R., Degezelle A., Maroutian T., Brubach J.-B., Demange V., Largeau L., Verseils M., Matzen S., Agnus G. (2023). Ferroelectric
ZrO_2_ phases from infrared spectroscopy. J. Mater. Chem. C..

[56] Dongare M. K., Malshe K., Gopinath C. S., Murwani I. K., Kemnitz E. (2004). Oxidation
activity and 18O-isotope exchange behavior of nickel oxide-stabilized
cubic zirconia. J. Catal..

[57] Ibrahim A., Fakeeha A., Abasaeed A., Al-Fatesh A. (2021). Dry Reforming
of Methane Using Ni Catalyst Supported on ZrO2: The Effect of Different
Sources of Zirconia. Catalysts.

[58] Ni J., Leng W., Mao J., Wang J., Lin J., Jiang D., Li X. (2019). Tuning electron density of metal
nickel by support defects in Ni/ZrO2 for selective hydrogenation of
fatty acids to alkanes and alcohols. Appl. Catal.,
B.

[59] Hoang D., Lieske H. (1994). Effect of hydrogen
treatments on ZrO2 and Pt/ZrO2 catalysts. Catal.
Lett..

[60] Korpelin V., Melander M. M., Honkala K. (2022). Reducing the
irreducible: Dispersed
metal atoms facilitate reduction of irreducible oxides. J. Phys. Chem. C.

[61] Kikas A., Aarik J., Kisand V., Kooser K., Käämbre T., Mändar H., Uustare T., Rammula R., Sammelselg V., Martinson I. (2007). Effect of phase composition on X-ray absorption spectra
of ZrO2 thin films. J. Electron Spectrosc. Relat.
Phenom..

[62] Christensen A., Carter E. A. (1998). First-principles
study of the surfaces of zirconia. Phys. Rev.
B.

[63] Wang Y., Li L., Li G., Zhao Q., Wu X. s., Wang Y., Sun Y., Hu C. (2023). Synergy of Oxygen Vacancies and Ni0 Species to Promote
the Stability of a Ni/ZrO2 Catalyst for Dry Reforming of Methane at
Low Temperatures. ACS Catal..

[64] Li B., Yuan X., Li B., Wang X. (2020). Impact of pore structure
on hydroxyapatite supported nickel catalysts (Ni/HAP) for dry reforming
of methane. Fuel Process. Technol..

[65] Li X., Shao Y., Zhang S., Wang Y., Xiang J., Hu S., Xu L., Hu X. (2021). Pore diameters of Ni/ZrO2 catalysts
affect properties of the coke in steam reforming of acetic acid. Int. J. Hydrogen Energy.

[66] Schneider D., Mehlhorn D., Zeigermann P., Kärger J., Valiullin R. (2016). Transport properties of hierarchical
micro–mesoporous
materials. Chem. Soc. Rev..

