# Supplementary material for: Milling-Induced Defects in Ni/Zirconia Catalysts for Enhancing Catalytic Activity in Dry Methane Reforming
Source: J Phys Chem C Nanomater Interfaces. 2026 Mar 4;130(11):4141–52. doi: 10.1021/acs.jpcc.5c08218 (PMC13007023; doi:10.1021/acs.jpcc.5c08218)
Supplement: Supplementary file 1 [file jp5c08218_si_001.pdf]

SUPPLEMENTARY INFORMATION

Milling-Induced Defects in Ni/Zirconia Catalysts for Enhancing Catalytic Activity in Dry Methane Reforming

Joanna Elzbieta Olszowka[1*], Volodymyr Sydorchuk,[1] Karolina Simkovicova,[1] Mehran Sajad,[1] Guillaume Clet,[2] Michal Horacek,[3] Graham King,[4] Jan Pasztor,[5] Stefan Vajda[1]

[1] Department of Nanocatalysis, J. Heyrovsky Institute of Physical Chemistry, Czech Academy of Sciences, 182 23 Prague, Czech Republic

[2] Université de Caen Normandie, ENSICAEN, CNRS, LCS, 14000 Caen, France

[3] Department of Molecular Electrochemistry and Catalysis, J. Heyrovsky Institute of Physical Chemistry, Czech Academy of Sciences, 182 23 Prague, Czech Republic

[4] Canadian Light Source, Saskatoon, SK S7N 2V3, Canada

[5] NICOLET CZ, 149 00 Prague, Czech Republic

Email of the corresponding author: joanna.olszowka@jh-inst.cas.cz

Table of Content:

Supplementary data on ex-situ characterization...S3

Table S1. Lattice and microstructural parameters for all ZrO_2 samples.................................S3

Figure S1. Atomic PDF for ZrO_2 supports and commercial ZrO_2 samples in the low-r and high-r ranges...S3

Figure S2. SEM micrographs of the prepared ZrO_2 support samples together with a commercial ZrO_2 reference..S4

Figure S3. Porosity analysis: a) nitrogen adsorption-desorption isotherms, and b) pore size distribution curves of the ZrO_2 support samples and commercial ZrO_2..................................S5

Figure S4. ATR-FTIR spectra of the support materials acquired in the middle infrared region; a) averaged spectra in the region 1850-800 cm^{-1}; b) averaged spectra in the region 1200-400 cm^{-1}, measured at room temperature in vacuum S6

Figure S5. Raman spectra of support materials measured at room temperature in vacuum....S7

Table S2. The Raman spectroscopy bands of monoclinic and tetragonal ZrO_2.......................S8

Details on catalytic data measured with the Ni-ZrO$_2$ assembly.............................S8

Table S3. CH_4 and CO_2 conversion on ZrO_2 support materials in DMR reaction at 550, 600, and 650 °C. S8

Figure S6. Conversion of CH_4 (empty circle) and CO_2 (filled circle) as a function of time on stream and temperature over Ni/ZrO$_2$_HT (gray), Ni/ZrO$_2$_HT_M400 (red), Ni/ZrO$_2$_HT_M450 (green), Ni/ZrO$_2$_HT_M500 (blue) catalysts...S9

Table S4. A comparison between previous reports on the Ni/ZrO$_2$ catalyst under DMR conditions and the current study……………………………………………………………..S10

Table S5. A comparison of spectroscopic benchmarks of ZrO_2 supports and Ni/ZrO$_2$ catalysts and the current study…………………………………………………………………S11

Figure S7. XRD pattern of the spent Ni/ZrO$_2$_HT_M450..S12

In situ characterization of the zirconia support……………………………………….S13

Figure S8. Diffuse reflectance infrared spectra in the far region measured during a heating procedure in argon for samples a) ZrO$_2$_HT, b) ZrO$_2$_HT_M400, c) ZrO$_2$_HT_M450, d) ZrO$_2$_HT_M500, and e) ZrO_2 commercial for comparison..S13

Figure S9. Hydrogen consumption curves recorded in the Raman cell during reduction of the selected Ni-ZrO$_2$ assemblies (NiO/ZrO$_2$_HT, NiO/ZrO$_2$_HT_M400, NiO/ZrO$_2$_HT_M450) and bare ZrO$_2$_HT_M450 support for comparison...S14

References...S15

Supplementary data on ex-situ characterization

Table S1. Lattice and microstructural parameters (average grain size D and microstrain concentration <e>) for all ZrO_2 samples.

Sample	Lattice parameters		Microstructural parameters
ZrO_2_HT	a = 5.150(2) Å	c = 5.320(2) Å	<e>=0.0045
	b = 5.212(2) Å	β = 99.36(1)°	D = 12.9 nm
	V =140.9(1) Å^3		
ZrO_2_HT_M400	a = 5.144(2) Å	c = 5.323(2) Å	<e> =0.0047
	b = 5.194(3) Å	β = 99.33(2)°	D = 12.2nm
	V =140.4(2) Å^3		
ZrO_2_HT_M450	a = 5.148(2) Å	c = 5.318(2) Å	<e>= 0.0060
	b = 5.209(2) Å	β = 99.29(2)°	D = 10.4 nm
	V =140.7(2) Å^3		
ZrO_2_HT_M500	a = 5.138(6) Å	c = 5.354(6) Å	<e>= 0.0166
	b = 5.191(6) Å	β = 98.02(4)°	D = 3.4 nm
	V =141.4(5) Å^3		

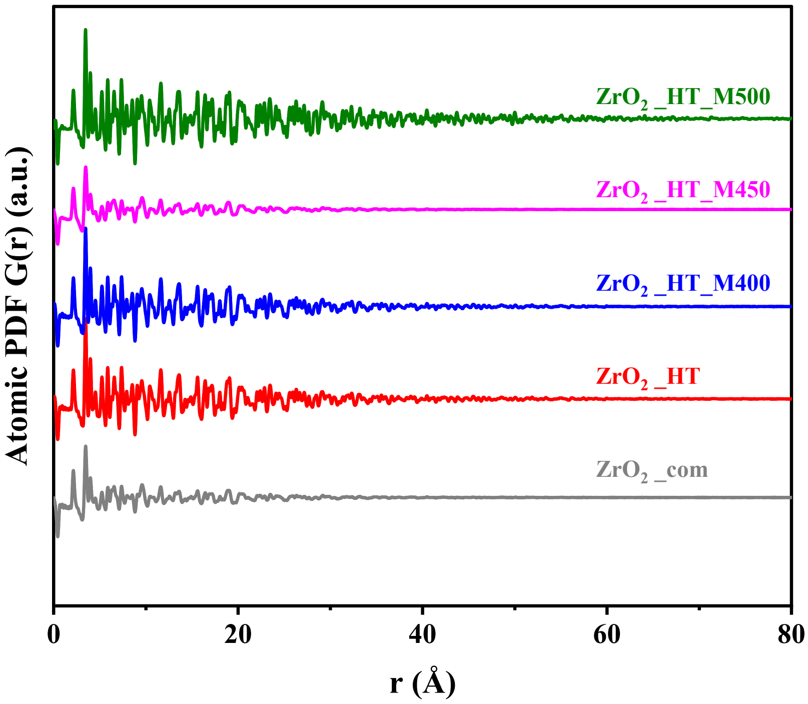

Figure S1. Atomic PDF for ZrO_2 supports and commercial ZrO_2 samples in the low-r and high-r ranges showing the level of distortion induced by milling.

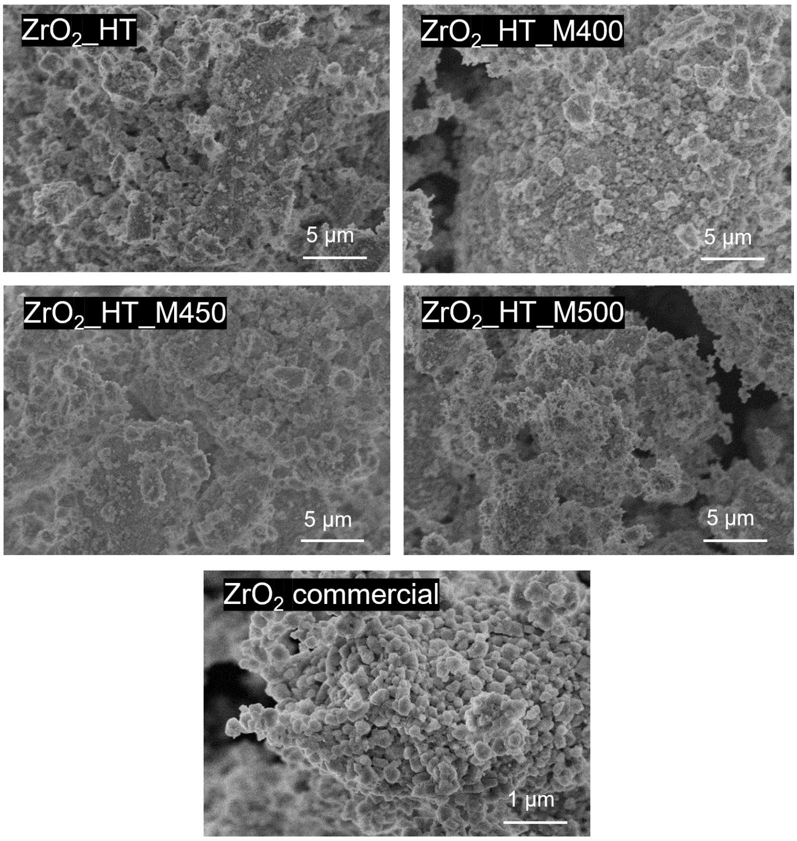

Figure S2. SEM micrographs of the prepared ZrO_2 support samples together with a commercial ZrO_2 reference, showing the effect of milling on the zirconia structure with visible signs of agglomeration.

a)

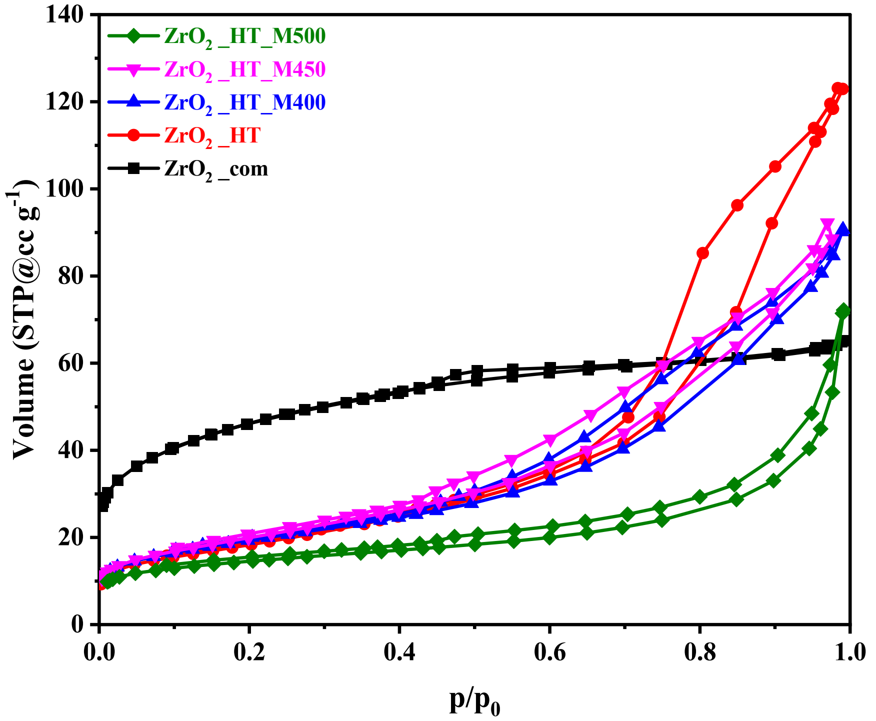

b)

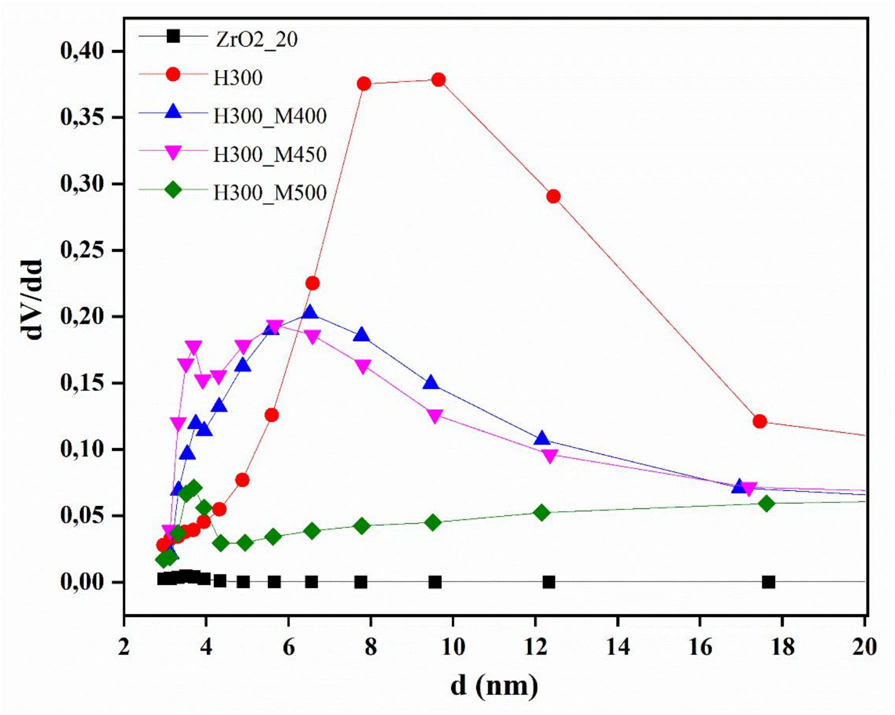

Figure S3. Porosity analysis: a) nitrogen adsorption-desorption isotherms, and b) pore size distribution curves of the ZrO_2 support samples and commercial ZrO_2, showing that milling of zirconia influences textural parameters of the materials.

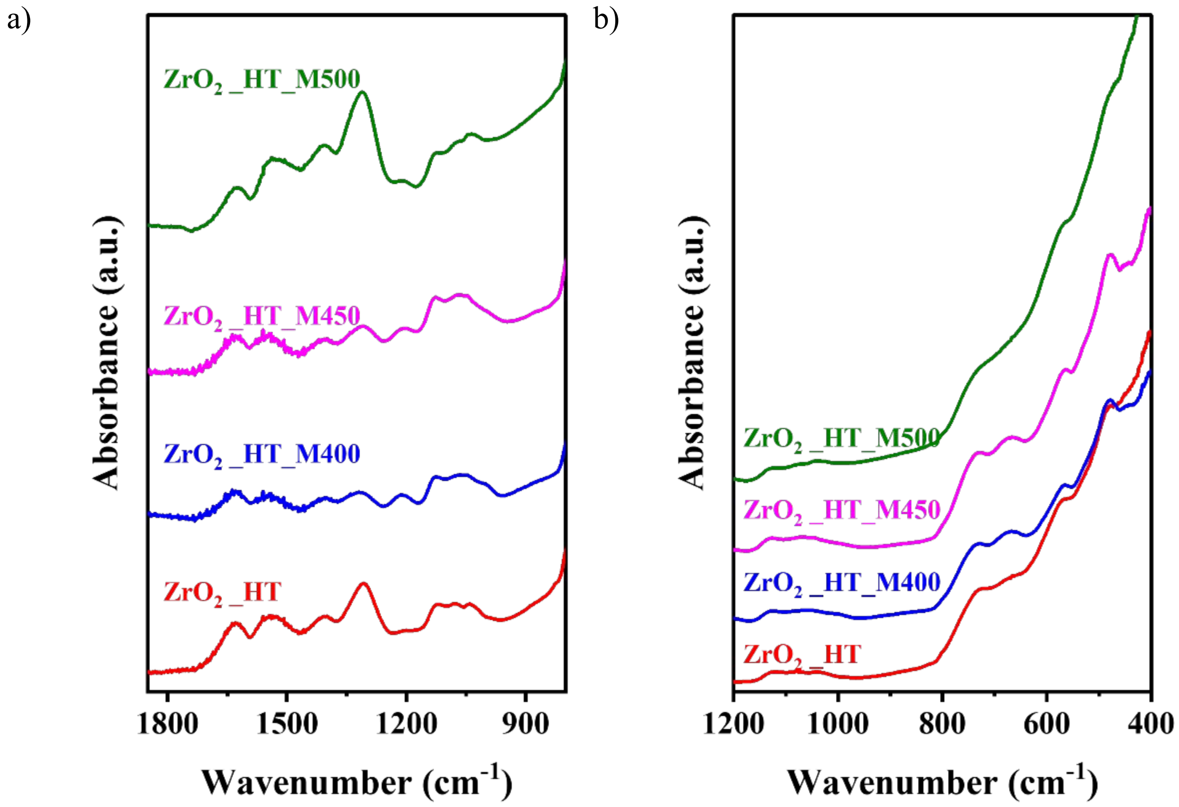

Figure S4. ATR-FTIR spectra of the support materials acquired in the middle infrared region; a) averaged spectra in the region 1850-800 cm^{-1}; b) averaged spectra in the region 1200-400 cm^{-1}, measured at room temperature in vacuum.

Attenuated total reflectance Fourier transform infrared spectroscopy (ATR-FTIR) spectra shows two regions: 1850-800 cm^{-1} associated mainly with surface adsorbates (Figure S4a), and 1200-400 cm^{-1} showing the lattice vibrations of Zr-O (Figure S4b). The bands at ca. 1630, 1540, 1405, 1310, 1210, 1126, 1070, and 1039 cm^{-1} may be attributed to nitrate species[1]. Some nitrates could indeed remain trapped in the material due to the low calcination temperature. The various relative intensities of these bands on the samples indicate different nitrate coordinations (linear, bidentate, or bridged), with the spectra separating into two groups of similar behavior. The spectrum of ZrO_2_HT_M400 resembles ZrO_2_HT_M450, while ZrO_2_HT_500 resembles ZrO_2_HT. This implies that while milling induces a rearrangement of the crystal lattice at the surface, the nitrates remaining from the synthesis protocol could also be affected.

The other most prominent feature at 480 cm^{-1} (see Figure S4b) is characteristic of the deformation vibration of Zr-O [2]. The peak increased significantly after the milling (samples ZrO_2_HT_M400 and ZrO_2_HT_M450), but with a further increase in milling intensity, it almost completely disappears in the sample ZrO_2_HT_M500. This suggests that prolonged milling leads to partial structural destruction.

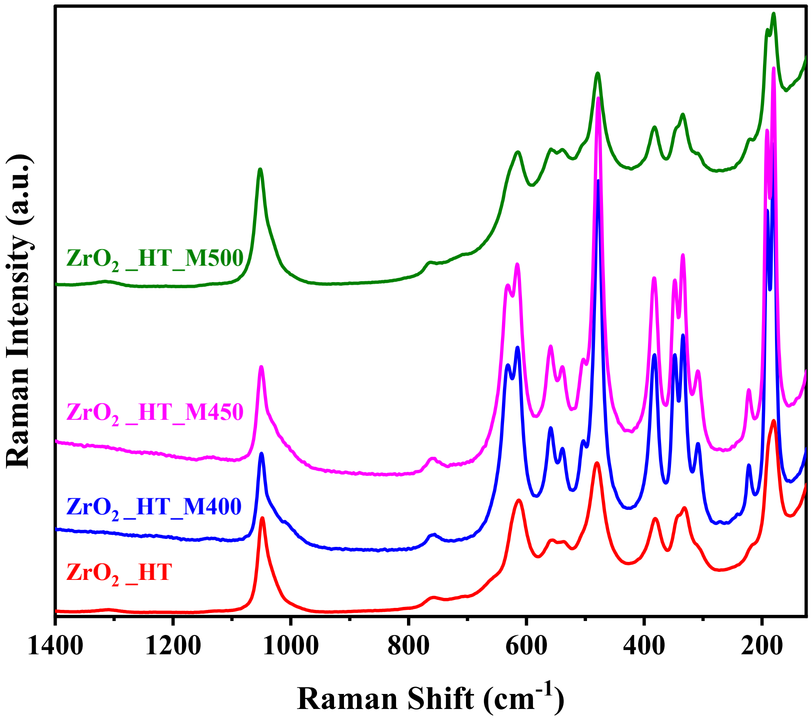

Figure S5. Raman spectra of support materials measured at room temperature in vacuum.

In the Raman spectra (Figure S5), the bands corresponding to the monoclinic structure of ZrO_2 are evident in all samples [3]. The list of the characteristic shifts of monoclinic and tetragonal ZrO_2 can be found in Table S2.

Additional bands are also observed around 1045 cm^{-1} with a smaller band at 755 cm^{-1}. These bands are found in many nitrate-containing salts[4]. This confirms that nitrate impurities remain after synthesis, as indicated above by the ATR-FTIR data.

The spectrum of the hydrothermal sample, ZrO_2_HT, contains only bands related to the monoclinic phase. Nevertheless, compared to the ZrO_2_HT sample, the ZrO_2_HT_M400 and ZrO_2_HT_M450 samples exhibit splitting of several bands (625, 340, and 180 cm^{-1}) into doublets, and the bands are sharper and more intense. All three doublets belong to the monoclinic phase, and their second components were already present on the spectrum of ZrO_2_HT as shoulders. This indicates a higher degree of crystal order of the M400 and M450 samples. The spectrum of the ZrO_2_HT_M500 sample is less resolved with broader and less intense bands due to partial amorphization of ZrO_2, but it does not contain bands related to any other ZrO_2 phase except monoclinic, which is in good agreement with the PDF results.

Table S2. The Raman spectroscopy bands of monoclinic and tetragonal ZrO_2 [5].

Monoclinic [cm^{-1}]	Tetragonal [cm^{-1}]
90	139
174	
211	
230	260
300	300
321	
370	
461	461
523	
546	
620	640

Details on catalytic data measured with the Ni-ZrO_2 assembly

Table S3. CH_4 and CO_2 conversion on ZrO_2 support materials in DMR reaction.

Catalyst	T (°C)	CH_4 conversion (%)	CO_2 conversion (%)
ZrO_2_HT	550	9	0
ZrO_2_HT_M400		7	0
ZrO_2_HT_M450		10	0
ZrO_2_HT_M500		8	0
commercial ZrO_2		0	0
ZrO_2_HT	600	2	1
ZrO_2_HT_M400		5	3
ZrO_2_HT_M450		2	2
ZrO_2_HT_M500		5	3
commercial ZrO_2		0	0
ZrO_2_HT	650	3	1
ZrO_2_HT_M400		5	3
ZrO_2_HT_M450		2	1
ZrO_2_HT_M500		5	2
commercial ZrO_2		0	0

*values are presented as an average over 1h at the given temperature

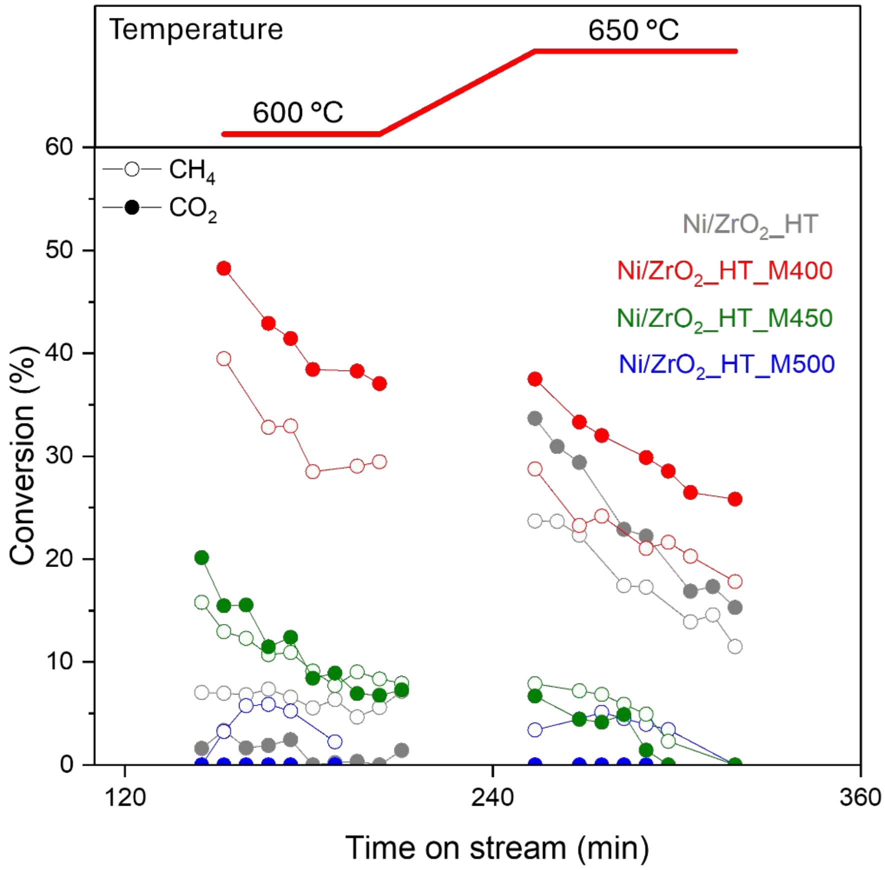

Figure S6. Conversion of CH_4 (empty circle) and CO_2 (filled circle) as a function of time on stream and temperature over Ni/ZrO_2_HT (gray), Ni/ZrO_2_HT_M400 (red), Ni/ZrO_2_HT_M450 (green), Ni/ZrO_2_HT_M500 (blue) catalysts. Note that during the change of temperature (10°C/min), the catalytic performance is excluded from the graph.

Table S4. A comparison between previous reports on the Ni/ZrO_2 catalyst under DMR conditions and the current study.

Catalyst	T (°C)	CH_4 conversion (%)	CO_2 conversion (%)	Ref.
Ni/ZrO_2-FUAH	850	98	95	[6]
5%Ni/ZrO_2	700	58	42	[7]
5%$Ni/CeZrO_2$	700	70	54	[7]
10%$Ni/CeZrO_2$	700	74	55	[7]
Ni/3Zr/MgO	750	80	85	[8]
Ni/ZrO_2 (RC)	600	68	67	[9]
Ni/ZrO_2 (RC)	800	65	67	[9]
10%Ni10%ZrO_2/Al_2O_3	620	54	50	[10]
2.5%Ni nanosheet/ZrO_2	800	46	60	[11]
10%Ni/m-ZrO_2-Al_2O_3	700	60	78	[12]
3%Ni/ZrO_2 treated with N_2	700	63	63	[13]
Ni/ZrO_2	750	80	88	[14]
5Ni15YZr	700	56	64	[15]
5Ni3Sr15YZr	700	67	80	[15]
Ni/ZrO_2-B	750	77	70	[16]
3NZH	700	70	80	[13]
Ni/ZrO_2_HT_M400	600	29	39	This work

Table S5. A comparison of spectroscopic benchmarks of ZrO_2 supports and Ni/ZrO_2 catalysts and the current study

Material	Raman	Operando Raman	FTIR	EPR	Ref.
Ni/ZrO_2	Monoclinic (m) and tetragonal (t) ZrO_2	Not studied	Not studied	Not studied	[17]
Ni/ZrO_2	m-ZrO_2 at RT	Not studied	In situ under oxidative and reductive conditions	Not studied	[18]
ZrO_2 prepared by the sol-gel method	m- and amorphous (a) ZrO_2	Not studied	Ex situ m- and a-ZrO2	Stability of OV under laser crystallization	[19]
m-ZrO_2 and compacted	m-ZrO_2	Not studied	Vibrational modes of Zr-O	Formation and removal of OV and Zr^{3+} during calcination	[20]
m-ZrO_2 shifts after calcination	m-ZrO_2	Not studied	Vibrational modes of Zr-O	Not studied	[21]
t-ZrO_2	t-ZrO_2 shifts towards m-ZrO_2	Not studied	Not studied	Formation of OV, Zr^{3+}	[22]
t-ZrO_2	m- and t-ZrO_2 mixed bands	Not studied	Not studied	Not studied	[23]
Yttria-stabilised zirconia (YSZ)	Raman bands of the YSZ after milling in water	Not studied	Not studied	Not studied	[24]
m-ZrO_2	m- and t-ZrO_2 mixed bands after milling in ethanol	Not studied	vibrational modes of Zr-O	Not studied	[25]
t-ZrO_2	t-ZrO_2	Not studied	Not studied	Not studied	[26]
Ni/ZrO_2 after HT/milling without high-temperature calcination	Raman shifts of monoclinic ZrO_2	m- and t-ZrO_2 mixed bands under DMR	In situ under exposure to 650 °C	OV, Zr^{3+} before and after DMR	This work

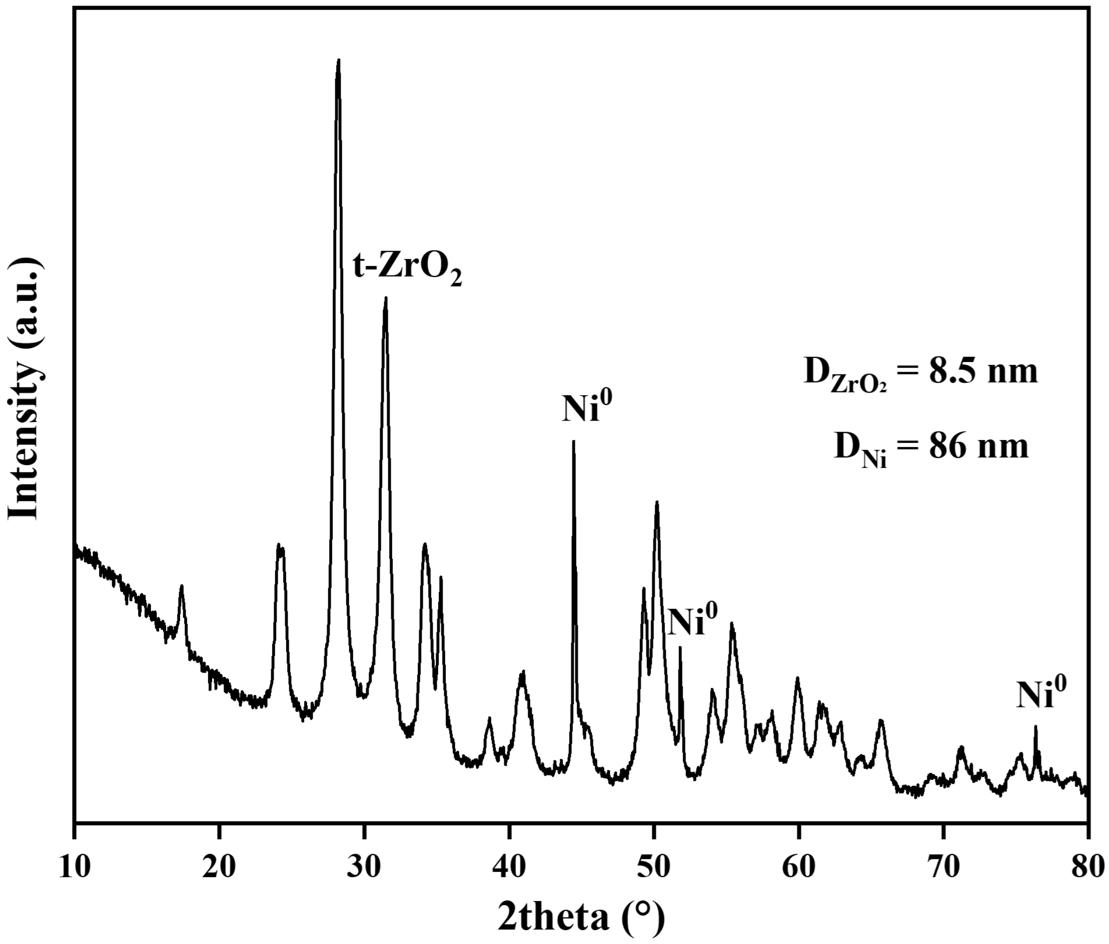

Figure S7. XRD pattern of the spent Ni/ZrO_2_HT_M450

In situ characterization of the zirconia support

Far Infrared in-situ characterization – temperature changes in argon

The diffuse reflectance measurements in the far IR region in Ar flow were performed on the ZrO_2_HT, ZrO_2_HT_H400, ZrO_2_HT_H450, and ZrO_2_HT_H500, as shown in Figure S7.

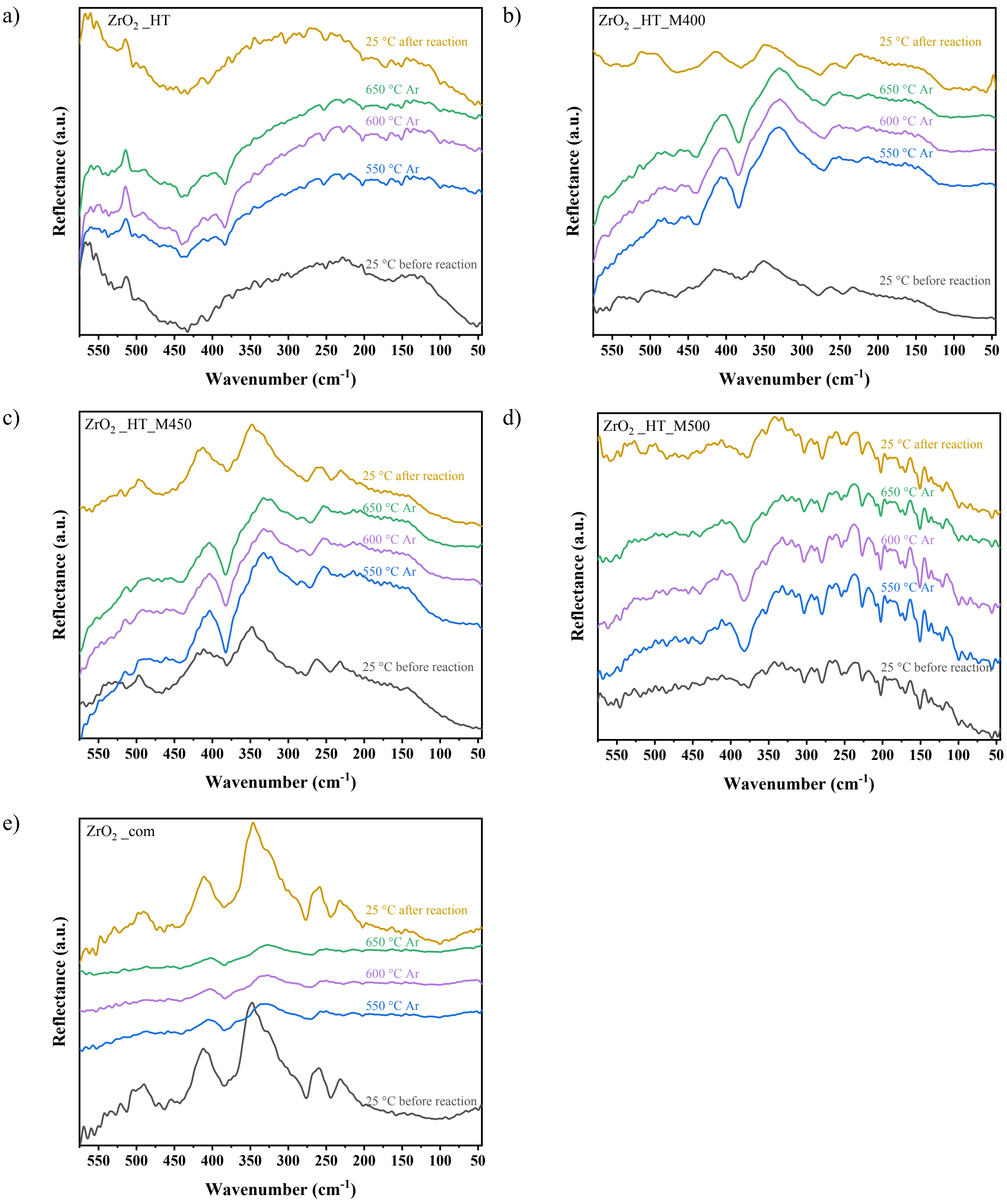

Figure S8. Diffuse reflectance infrared spectra in the far region measured during a heating procedure in argon for samples a) ZrO_2_HT, b) ZrO_2_HT_M400, c) ZrO_2_HT_M450, d) ZrO_2_HT_M500, and e) ZrO_2 commercial for comparison, showing temperature response of the materials.

In the spectra, one can identify the characteristic band positions of tetragonal ZrO_2 at 135, 358, and 471 cm^{-1} [27] and monoclinic ZrO_2 at 230, 262, 348, 375, 411, 444, 452, 494, 524, and 541 cm^{-1}.[20, 28] At the same time, the bands that can be attributed to the tetragonal phase are of low intensity. Therefore, it is difficult to judge the presence of a tetragonal phase on this basis.

The sets of spectra show a similarity between ZrO_2_HT and ZrO_2_HT_M500, and between ZrO_2_HT_M400 and ZrO_2_HT_M450. The latter group also exhibits stronger bands, possibly pointing to a more crystalline and ordered structure.

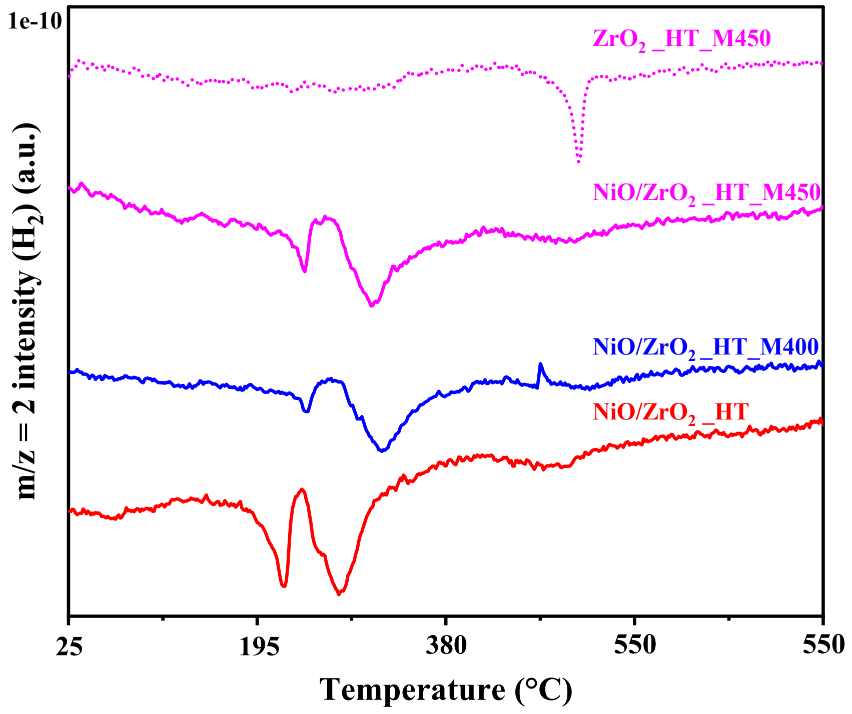

Figure S9. Hydrogen consumption curves recorded in the Raman cell during reduction of the selected Ni-ZrO_2 assemblies (NiO/ZrO_2_HT, NiO/ZrO_2_HT_M400, NiO/ZrO_2_HT_M450) and bare ZrO_2_HT_M450 support for comparison, showing a shift in reduction temperature for catalysts with milled supports.

The spectrum of the sample ZrO_2_HT mainly contains bands of the monoclinic phase and, possibly, several bands of the tetragonal phase (Figure S7a). However, the above-mentioned results of XRD and Raman spectroscopy indicate the prevalence of monoclinic ZrO_2. Additionally, the band at 524 cm^{-1} is the strongest and the most intense. The heating of this sample causes some of the bands to become more pronounced, mainly at 230, 262, 264, 444, 494, and 524 cm^{-1}. Further heating to 650 °C does not cause any additional changes. After cooling down to 20 °C, the spectrum is identical to the one before the reaction.

For sample ZrO_2_HT_M400, the band at 524 cm^{-1} has a very low intensity, while there are the most intense and broad bands are at 411 and 348 cm^{-1} (Figure S7b). After heating in argon, they become more intense and broader. Besides, the positions of their maximum are shifted to 400 and 330 cm^{-1}, respectively. Upon cooling down back to room temperature, a spectrum similar

spectrum ZrO_2_HT_H400, with the stronger monoclinic ZrO_2 bands. Upon heating the sample in argon, the bands of monoclinic ZrO_2 at 444 and 452 cm^{-1} get stronger. Cooling down this sample to 20 °C does not change the spectrum compared to the one before the reaction, except for stronger bands at 518 and 541 $cm^{-1,}$ which also belong to the monoclinic phase. In addition, before heating and after cooling, a sharper band at 494 cm^{-1} appears in the spectrum of the sample ZrO_2_HT_M450 (Figure S7c). Thus, it can be stated that the pure monoclinic phase is preserved when the sample is heated in argon to 650 °C and then cooled down to room temperature. The spectra for sample ZrO_2_HT_H500 (Figure S7d) are noisier than the rest of the samples. All these spectra (before heating, after heating, after next cooling) are almost the same and contain bands of approximately the same intensity, but the main bands of monoclinic and tetragonal ZrO_2 can still be recognized. For example, bands at 235 and 358 cm^{-1}, which are characteristic of the tetragonal phase, are detectable in the spectra (Figure S7d). The spectra at higher temperatures (500, 550, and 650 °C) resemble the spectra of ZrO_2_HT, and when cooled down to 20 °C, the spectrum returns to have the same features as before the reaction.

Therefore, hydrothermal and post-milled samples contain an almost pure monoclinic phase after heating and cooling in argon in accordance with spectroscopic data. However, milling introduces changes in its structure, which causes a different set of bands and their intensities in the spectra compared to the hydrothermal sample. Notable is the appearance of a broad band between 390-440 cm^{-1}, asymmetrical towards higher wavenumbers, which may include a band at 434 cm^{-1}, in the spectra of the samples ZrO_2_HT_M400 and ZrO_2_HT_M450. This band at 434 cm^{-1} is ascribed to surface phonons. [28].

References for Supplementary Information

(1) Hadjiivanov, K. I. Identification of Neutral and Charged N x O y Surface Species by IR Spectroscopy. *Catalysis Reviews* **2000**, *42* (1-2), 71-144. DOI: 10.1081/CR-100100260.
(2) Kataoka, S.; Sue, K. Enhanced Solubility of Zirconium Oxo Clusters from Diacetoxyzirconium(IV) Oxide Aqueous Solution as Inorganic Extreme-Ultraviolet Photoresists. *European Journal of Inorganic Chemistry* **2022**, *2022* (12). DOI: 10.1002/ejic.202200050.
(3) Pokratath, R.; Lermusiaux, L.; Checchia, S.; Mathew, J. P.; Cooper, S. R.; Mathiesen, J. K.; Landaburu, G.; Banerjee, S.; Tao, S.; Reichholf, N. An amorphous phase precedes crystallization: unraveling the colloidal synthesis of zirconium oxide nanocrystals. *ACS nano* **2023**, *17* (9), 8796-8806. Rijckaert, H.; De Roo, J.; Van Zele, M.; Banerjee, S.; Huhtinen, H.; Paturi, P.; Bennewitz, J.; Billinge, S. J.; Bäcker, M.; De Buysser, K. Pair distribution function analysis of ZrO2 nanocrystals and insights in the formation of ZrO2-YBa2Cu3O7 nanocomposites. *Materials* **2018**, *11* (7), 1066.
(4) Zapata, F.; García-Ruiz, C. The discrimination of 72 nitrate, chlorate and perchlorate salts using IR and Raman spectroscopy. *Spectrochimica Acta Part A: Molecular and Biomolecular Spectroscopy* **2018**, *189*, 535-542. DOI: https://doi.org/10.1016/j.saa.2017.08.058.
(5) Keramidas, V. G.; White, W. B. Raman Scattering Study of the Crystallization and Phase Transformations of ZrO2. *Journal of the American Ceramic Society* **2006**, *57* (1), 22-24. DOI: 10.1111/j.1151-2916.1974.tb11355.x. Thakur, M.; Vij, A.; Singh, F.; Rangra, V. S. Spectroscopic studies of metastable tetragonal ZrO2 nanocrystals. *Spectrochimica Acta Part A: Molecular and Biomolecular Spectroscopy* **2024**, *305*, 123495.
(6) Li, W.; Zhao, Z.; Wang, G. Modulating morphology and textural properties of ZrO2 for supported Ni catalysts toward dry reforming of methane. *AIChE Journal* **2017**, *63* (7), 2900-2915.
(7) Sophiana, I. C.; Iskandar, F.; Devianto, H.; Nishiyama, N.; Budhi, Y. W. Coke-resistant

Ni/CeZrO2 catalysts for dry reforming of methane to produce hydrogen-rich syngas. *Nanomaterials* **2022**, *12* (9), 1556.
(8) Chatla, A.; Almanassra, I. W.; Kallem, P.; Atieh, M. A.; Alawadhi, H.; Akula, V.; Banat, F. Dry (CO2) reforming of methane over zirconium promoted Ni-MgO mixed oxide catalyst: Effect of Zr addition. *Journal of CO2 Utilization* **2022**, *62*, 102082.
(9) Ibrahim, A. A.; Fakeeha, A. H.; Lanre, M. S.; Al-Awadi, A. S.; Alreshaidan, S. B.; Albaqmaa, Y. A.; Adil, S. F.; Al-Zahrani, A. A.; Abasaeed, A. E.; Al-Fatesh, A. S. The effect of calcination temperature on various sources of ZrO2 supported Ni catalyst for dry reforming of methane. *Catalysts* **2022**, *12* (4), 361.
(10) Sumarasingha, W.; Supasitmongkol, S.; Phongaksorn, M. The effect of ZrO2 as different components of Ni-based catalysts for CO2 reforming of methane and combined steam and CO2 reforming of methane on catalytic performance with coke formation. *Catalysts* **2021**, *11* (8), 984.
(11) Tathod, A. P.; Hayek, N.; Shpasser, D.; Simakov, D. S.; Gazit, O. M. Mediating interaction strength between nickel and zirconia using a mixed oxide nanosheets interlayer for methane dry reforming. *Applied Catalysis B: Environmental* **2019**, *249*, 106-115.
(12) Wang, X.; Bai, X.; Guo, Y.; Liu, Q.; Ji, S.; Wang, Z. j. A nanoscale Ni/ZrO2 catalyst coated with Al2O3 for carbon dioxide reforming of methane. *Journal of Chemical Technology & Biotechnology* **2021**, *96* (2), 474-480.
(13) Zhang, M.; Zhang, J.; Wu, Y.; Pan, J.; Zhang, Q.; Tan, Y.; Han, Y. Insight into the effects of the oxygen species over Ni/ZrO2 catalyst surface on methane reforming with carbon dioxide. *Applied Catalysis B: Environmental* **2019**, *244*, 427-437.
(14) Zhang, M.; Yang, T.; Jiang, K.; Gao, Y.; Yang, J.; Liu, Z.; Han, Y. Rationally constructing metastable ZrO2 supported Ni catalysts for highly efficient and stable dry reforming of methane. *Applied Catalysis B: Environment and Energy* **2024**, *353*, 124102.
(15) Fakeeha, A. H.; Kurdi, A.; Al-Baqmaa, Y. A.; Ibrahim, A. A.; Abasaeed, A. E.; Al-Fatesh, A. S. Performance study of methane dry reforming on Ni/ZrO2 catalyst. *Energies* **2022**, *15* (10), 3841.
(16) Shao, J.; Li, C.; Fei, Z.; Liu, Y.; Zhang, J.; Li, L. MOFs-derived Ni@ ZrO2 catalyst for dry reforming of methane: Tunable metal-support interaction. *Molecular Catalysis* **2024**, *558*, 114028.
(17) Li, W.; Zhao, Z.; Ding, F.; Guo, X.; Wang, G. Syngas Production via Steam–CO2 Dual Reforming of Methane over LA-Ni/ZrO2 Catalyst Prepared by l-Arginine Ligand-Assisted Strategy: Enhanced Activity and Stability. *ACS Sustainable Chemistry & Engineering* **2015**, *3* (12), 3461-3476. DOI: 10.1021/acssuschemeng.5b01277.
(18) Pietrogiacomi, D.; Campa, M. C.; Pettiti, I.; Tuti, S.; Luccisano, G.; Ardemani, L.; Luisetto, I.; Gazzoli, D. Oscillatory Behaviour of Ni Supported on ZrO2 in the Catalytic Partial Oxidation of Methane as Determined by Activation Procedure. *Materials* **2021**, *14* (10), 2495.
(19) Benavides-Guerrero, J. A.; Gerlein, L. F.; Angel-Ospina, A. C.; Fourmont, P.; Bhattacharya, A.; Zirakjou, A.; Vaussenat, F.; Ross, C. A.; Cloutier, S. G. Room-temperature laser crystallization of oxygen vacancy-engineered zirconia for additive manufacturing. *Additive Manufacturing* **2025**, *111*, 104969. DOI: https://doi.org/10.1016/j.addma.2025.104969.
(20) Maczka, M.; Lutz, E. T. G.; Verbeek, H. J.; Oskam, K.; Meijerink, A.; Hanuza, J.; Stuivinga, M. Spectroscopic studies of dynamically compacted monoclinic ZrO_2. *Journal of physics and chemistry of solids* **1999**, *60* (12), 1909-1914.
(21) Cyriac, B. Zirconia: synthesis and characterization. In *Zirconia-New Advances, Structure, Fabrication and Applications*, IntechOpen, 2023.
(22) Matta, J.; Lamonier, J.-F.; Abi-Aad, E.; Zhilinskaya, E. A.; Aboukaïs, A. Transformation of tetragonal zirconia phase to monoclinic phase in the presence of Fe 3+ ions as probes: an

EPR study. *Physical Chemistry Chemical Physics* **1999**, *1* (21), 4975-4980.
(23) Zhao, L.; Zhao, J.; Wu, T.; Zhao, M.; Yan, W.; Zhang, Y.; Li, H.; Wang, Y.; Xiao, T.; Zhao, Y. Synergistic Effect of Oxygen Vacancies and Ni Species on Tuning Selectivity of Ni/ZrO(2) Catalyst for Hydrogenation of Maleic Anhydride into Succinic Anhydride and gamma-Butyrolacetone. *Nanomaterials (Basel)* **2019**, *9* (3). DOI: 10.3390/nano9030406 From NLM PubMed-not-MEDLINE.
(24) Jianxing, Z.; Zongyu, F.; Jianhui, S.; He, Z.; Juanyu, Y.; Ning, W.; Fang, C.; Lei, D.; Xiaowei, H. Crystal defects and phase transitions of nanocrystalline yttria-stabilised zirconia induced by high-energy ball milling. *Ceramics International* **2021**, *47* (12), 16432-16440. DOI: https://doi.org/10.1016/j.ceramint.2020.11.044.
(25) Shetty, S. J.; Kumar M, M.; George, A.; Rajendran, A.; SC, G.; P, V.; Sharda, T.; KG, R. Ball milling-induced evolution of structural, optical, and catalytic properties in nanocrystalline ZrO2. *Materials Research Express* **2025**, *12* (12), 125012.
(26) Luo, P.; Wang, J.; Rui, W.; Xu, R.; Kuai, Z.; Yang, D.; Wan, X.; Zhou, C.; Yang, Y.; Dai, Y. Ball-milling-induced phase transition of ZrO2 promotes selective oxidation of glycerol to dihydroxyacetone over supported PtBi bimetal catalyst. *Chemical Engineering Journal* **2023**, *467*, 143502. DOI: https://doi.org/10.1016/j.cej.2023.143502.
(27) El Boutaybi, A.; Cervasio, R.; Degezelle, A.; Maroutian, T.; Brubach, J.-B.; Demange, V.; Largeau, L.; Verseils, M.; Matzen, S.; Agnus, G.; et al. Ferroelectric ZrO_2 phases from infrared spectroscopy. *Journal of Materials Chemistry C* **2023**, *11* (32), 10931-10941. DOI: 10.1039/d3tc01985c.
(28) Zhang, H.; Liu, Y.; Zhu, K.; Siu, G.; Xiong, Y.; Xiong, C. Infrared spectra of nanometre granular zirconia. *Journal of Physics: Condensed Matter* **1999**, *11* (8), 2035-2042. DOI: 10.1088/0953-8984/11/8/016.
